# Dys-regulated phosphatidylserine externalization as a cell intrinsic immune escape mechanism in cancer

**DOI:** 10.1186/s12964-025-02090-6

**Published:** 2025-03-11

**Authors:** Rachael Pulica, Ahmed Aquib, Christopher Varsanyi, Varsha Gadiyar, Ziren Wang, Trevor Frederick, David C. Calianese, Bhumik Patel, Kenneth Vergel de Dios, Victor Poalasin, Mariana S. De Lorenzo, Sergei V. Kotenko, Yi Wu, Aizen Yang, Alok Choudhary, Ganapathy Sriram, Raymond B. Birge

**Affiliations:** 1 Department of Microbiology, Biochemistry and Molecular Genetics, Center for Cell Signaling, Rutgers New Jersey Medical School, 205 South Orange Ave, Newark, NJ 07103 USA; 2 Department of Cell Biology and Molecular Medicine, 185 South Orange Ave, Newark, NJ 07103 USA; 3 Collaborative Innovation Center of Hematology, State Key Laboratory of Radiation Medicine and Prevention, Cyrus Tang Medical Institute, Soochow University, Suzhou, China; 4 International Center for Public Health, Public Health Research Institute, Newark, NJ 07103 USA; 5 Department Biological, Chemical and Environmental Sciences, Wheaton College, 26 E Main St, Norton, MA 02766 USA

**Keywords:** Phosphatidylserine, P4 ATPase, Scramblases, PS receptors, Immune escape

## Abstract

The negatively charged aminophospholipid, phosphatidylserine (PS), is typically restricted to the inner leaflet of the plasma membrane under normal, healthy physiological conditions. PS is irreversibly externalized during apoptosis, where it serves as a signal for elimination by efferocytosis. PS is also reversibly and transiently externalized during cell activation such as platelet and immune cell activation. These events associated with physiological PS externalization are tightly controlled by the regulated activation of flippases and scramblases. Indeed, improper regulation of PS externalization results in thrombotic diseases such as Scott Syndrome, a defect in coagulation and thrombin production, and in the case of efferocytosis, can result in autoimmunity such as systemic lupus erythematosus (SLE) when PS-mediated apoptosis and efferocytosis fails. The physiological regulation of PS is also perturbed in cancer and during viral infection, whereby PS becomes persistently exposed on the surface of such stressed and diseased cells, which can lead to chronic thrombosis and chronic immune evasion. In this review, we summarize evidence for the dysregulation of PS with a main focus on cancer biology and the pathogenic mechanisms for immune evasion and signaling by PS, as well as the discussion of new therapeutic strategies aimed to target externalized PS. We posit that chronic PS externalization is a universal and agnostic marker for diseased tissues, and in cancer, likely reflects a cell intrinsic form of immune escape. The continued development of new therapeutic strategies for targeting PS also provides rationale for their co-utility as adjuvants and with immune checkpoint therapeutics.

**Supplementary Information:**

The online version contains supplementary material available at 10.1186/s12964-025-02090-6.

## Phospholipid asymmetry and phosphatidylserine (PS) externalization mechanisms

Eukaryotic cells are enveloped by a semipermeable lipid bilayer called the plasma membrane, or cell membrane, that functions as a physical barrier between the interior of the cell and the external environment. One of the most notable features of the plasma membrane is that the phospholipids are asymmetrically distributed across the bilayer. Anionic phospholipids phosphatidylserine (PS) and phosphatidylethanolamine (PE) are almost exclusively restricted to the inner leaflet, while the neutral phospholipid phosphatidylcholine (PC), along with cholesterol and neutral sphingolipid sphingomyelin (SM), are localized to the outer leaflet [1–3]. This topologic organization of phospholipids in the plasma membrane is a hallmark of viable and healthy cells.

**Fig. 1:**
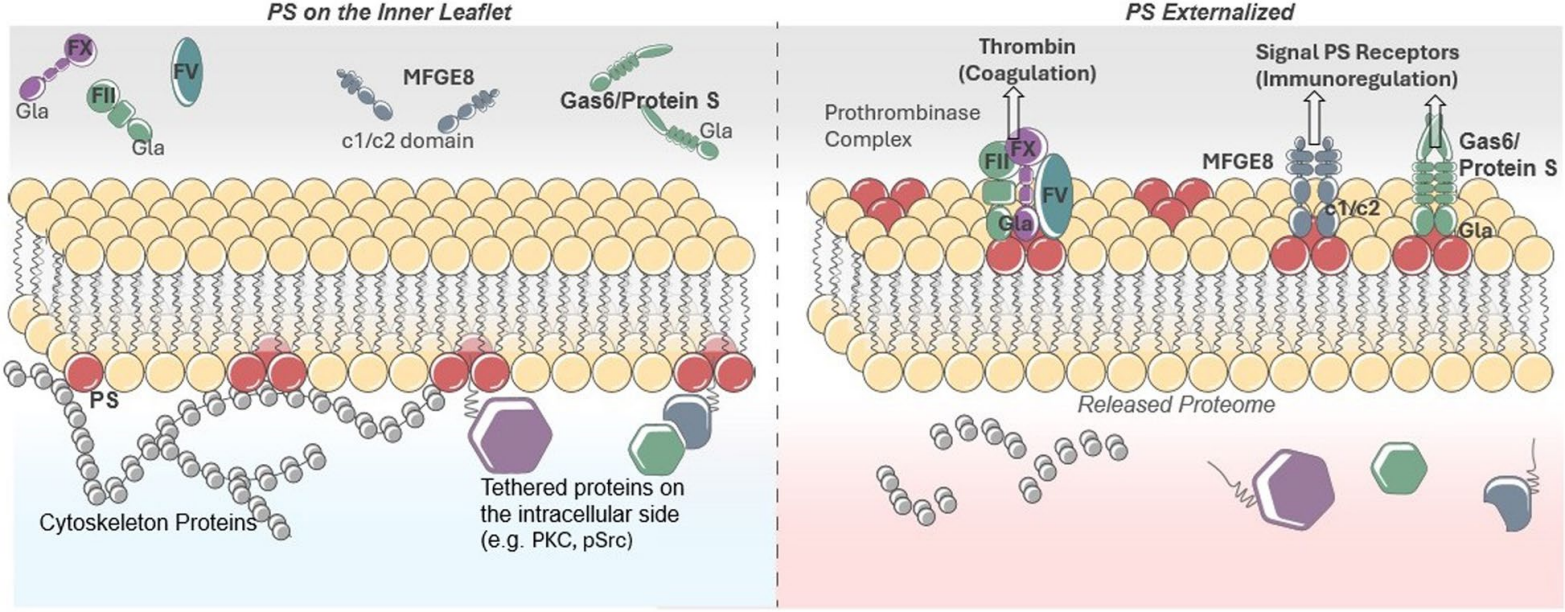
Intracellular and extracellular pools of PS. The proteomes of cells internalizing versus externalizing PS are unique from one another. When PS is on the inner side of the PM, it binds signaling proteins and cytoskeletal proteins. In contrast, on the exterior of the PM, coagulation factors and immunoregulatory molecules remain unbound. Upon PS externalization, the inner membrane becomes the released proteome, meaning that these signaling and cytoskeletal proteins are released from the PM. Meanwhile, on the external side of the cell, PS binds up coagulation factors and immunoregulatory molecules, resulting in the induction of their given pathways

The regulation of PS asymmetry, the subject of this review, is important for many essential structural and functional organizations of the cell membrane due to specific features of the ionic headgroup. Since PS (and PE) are acidic phospholipids, their asymmetric distribution at homeostasis renders the inner leaflet of the plasma membrane more acidic and negatively charged than the outer leaflet [4]. Such asymmetry of PS is paramount for critical biochemical properties of the membrane including inward bending of the membrane, caveola function, endocytosis, and vesicle trafficking, as well as maintenance of ionic channels and membrane potential [4]. Moreover, the negative charge is important for attracting many intracellular signaling proteins with focal stretches of positively charged basic amino acids that can electrostatically interact with PS on the inner surface, where PS acts as a scaffold to regulate enzymatic activity. Examples include signaling proteins that control proliferation and survival such as c-Src [5], Ras and Raf [6], Akt and PDK1 [7, 8], iPLA2 [9], nNOS [10], and PKC-γ [11], the extended family of Annexins that bind to PS in a calcium-dependent manner [12], as well as several signaling and structural proteins that control cytoskeletal organization and cellular organization such as Rac-1 [13], Vinculin [14, 15], and actin-binding proteins [16] (Fig. 1). Asymmetrically distributed PS can also control the physiological activation of transmembrane proteins such as receptor tyrosine kinases and integrins that recruit multicellular protein complexes once activated by tyrosine phosphorylation [17]. Since almost all of the PS (~ 98%) is restricted to the inner leaflet of the plasma membrane in healthy viable cells, the “homeostatic PS proteome” is virtually all comprised of cytosolic proteins that bind, localize, signal, and function from the inner leaflet of the plasma membrane to their cytosolic and cytoskeletal itineraries (Fig. 1). As noted again below, once PS is externalized, a priori a new set of extracellular proteins are recruited to the outer surface of the membrane.

### Regulation of PS biosynthesis

**Fig. 2:**
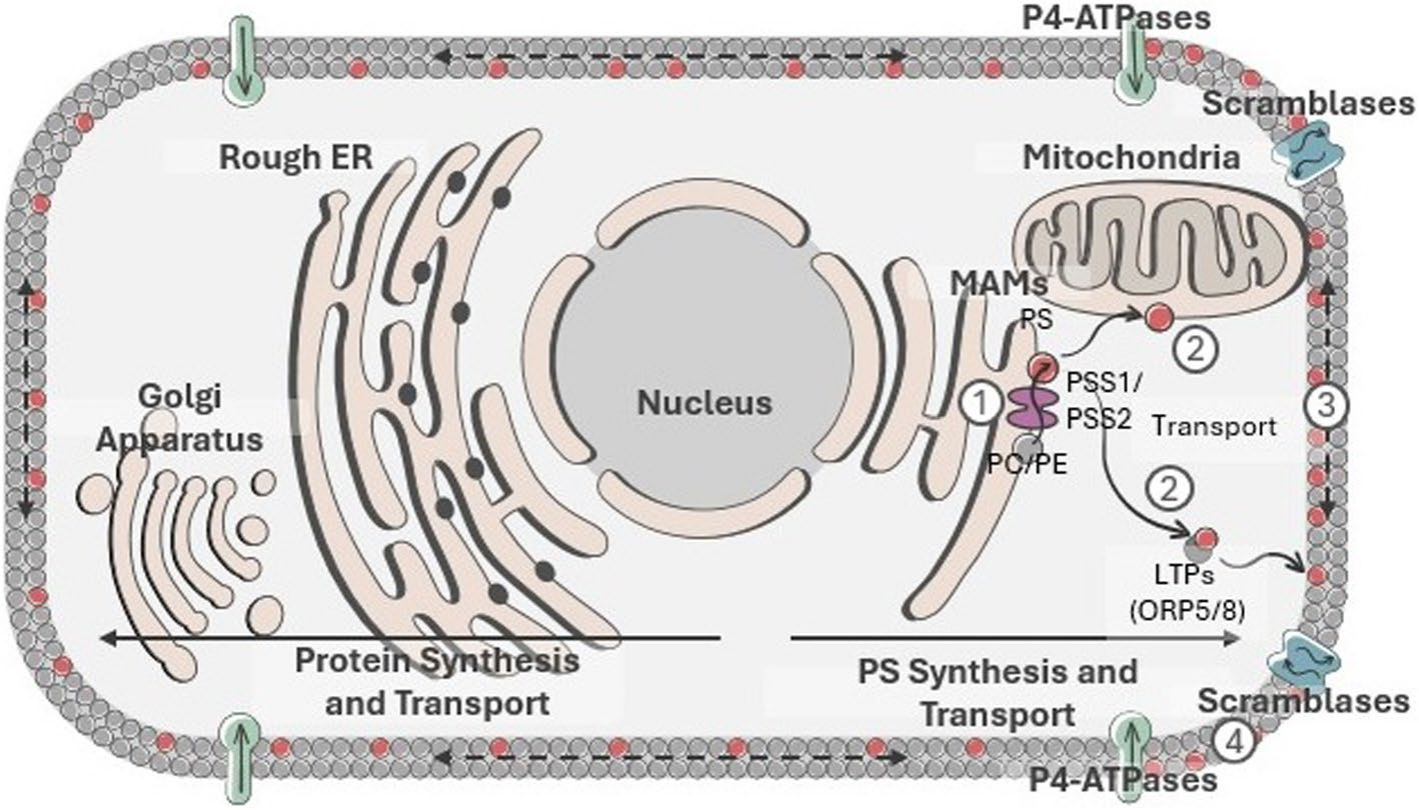
PS synthesis at the mitochondria-associated membranes (MAMs) and transport to the plasma membrane (PM). (1) PS is first synthesized via the PSS1/PSS2-mediated conversion of PC and PE to PS at the MAMs. (2) PS is then transported to the outer mitochondrial membrane and inner PM by lipid transfer proteins (e.g. ORP5/8). (3) Unrestricted lateral movement of PS then shifts it across the PM. (4) Once integrated in the PM, PS is externalized by scramblases and internalized by P4-ATPases.​ These arrangements are expected to restrict PS binding proteins that are destined for secretion to become trapped by intracellular pools for PS

At the biochemical and cell biological level, PS is first synthesized at mitochondrial-associated membrane (MAM) structures [4], the site where the rate-limiting enzymes Phosphatidylserine Synthetase 1 (PS-S1) and Phosphatidylserine Synthetase 2 (PS-S2) are localized. Subsequently, P4 type ATP-dependent lipid transport enzymes appear to first encounter de novo synthesized PS later at the trans-golgi network. Since both PS-S1 and PS-S2, the enzymes that catalyze PS biosynthesis from PE and PC respectively, are concentrated in MAMs and not the ER, intracellular PS appears to be largely excluded from the bulk of the rough endoplasmic reticulum [18, 19] (Fig. 2). The localization of PS-S1 and PS-S2 at MAMs is also important for the transport of PS into the mitochondria, where PS is converted into PE by PS decarboxylase, the latter a critical lipid for the function of the inner mitochondrial membrane and electron transport and ATP production [20]. Notably, the localization of PS-S1 and PS-S2 to the MAM structures appears to, at least partly, ensure that PS-binding proteins destined from (ribosomal) translation and translocation to the lumen of the ER and extracellular itineraries are not trapped and retained by binding to intracellular pools of PS. As such, proteins with such high-affinity PS binding domains destined for ER trafficking and secretion, such as PS receptors and proteins with Gamma carboxyglutamic acid (Gla) domains or C5/8-type C2 domains, are effectively transported to the secretory pathways as extracellular proteins since PS has a low concentration in the ER (Fig. 2). During the transfer of PS from the ER to the plasma membrane, which in part is facilitated by lipid transporter proteins such as ORP5 and ORP8 or by intracellular vesicles, PS may already be associated with proteins destined for the plasma membrane as has been reported for both the localization and activation of KRAS [21, 22].

Subsequently, once PS from directional flow or lipid transfer proteins reaches the plasma membrane, P4-ATPases continuously catalyze PS asymmetry by flipping lipids from the luminal side of internal membranes to the cytosolic surface such that when PS reaches the plasma membrane, it is almost exclusively localized inward towards the cytosolic surface. As noted above, such organization of PS is important to maintain the extracellular and intracellular PS-binding proteomes as separate entities. With respect to the distribution of PS on the internal side of the plasma membrane, although there are as many as 15 members of P4-ATPases in mammalian cells, and while redundancies in the specificities exist and have dynamic distributions [23], many of the P4-ATPase enzymes (including ATP11A, ATP11C, ATP8A1, ATP8A2, ATP8B4, ATP10A, and ATP8B2) can be localized at the plasma membrane via their association with CDC50A (as opposed to intracellular membranes without CDC50 association), and many have preferential specificity towards PS compared to PC and/or PE [24]. However, the observations that caspase cleavage of certain flippases (ATP11C and ATP11A) can produce constitutively externalized PS suggests some hierarchy may exist at the functional level, or they may have dominant positive effects.

**Fig. 3:**
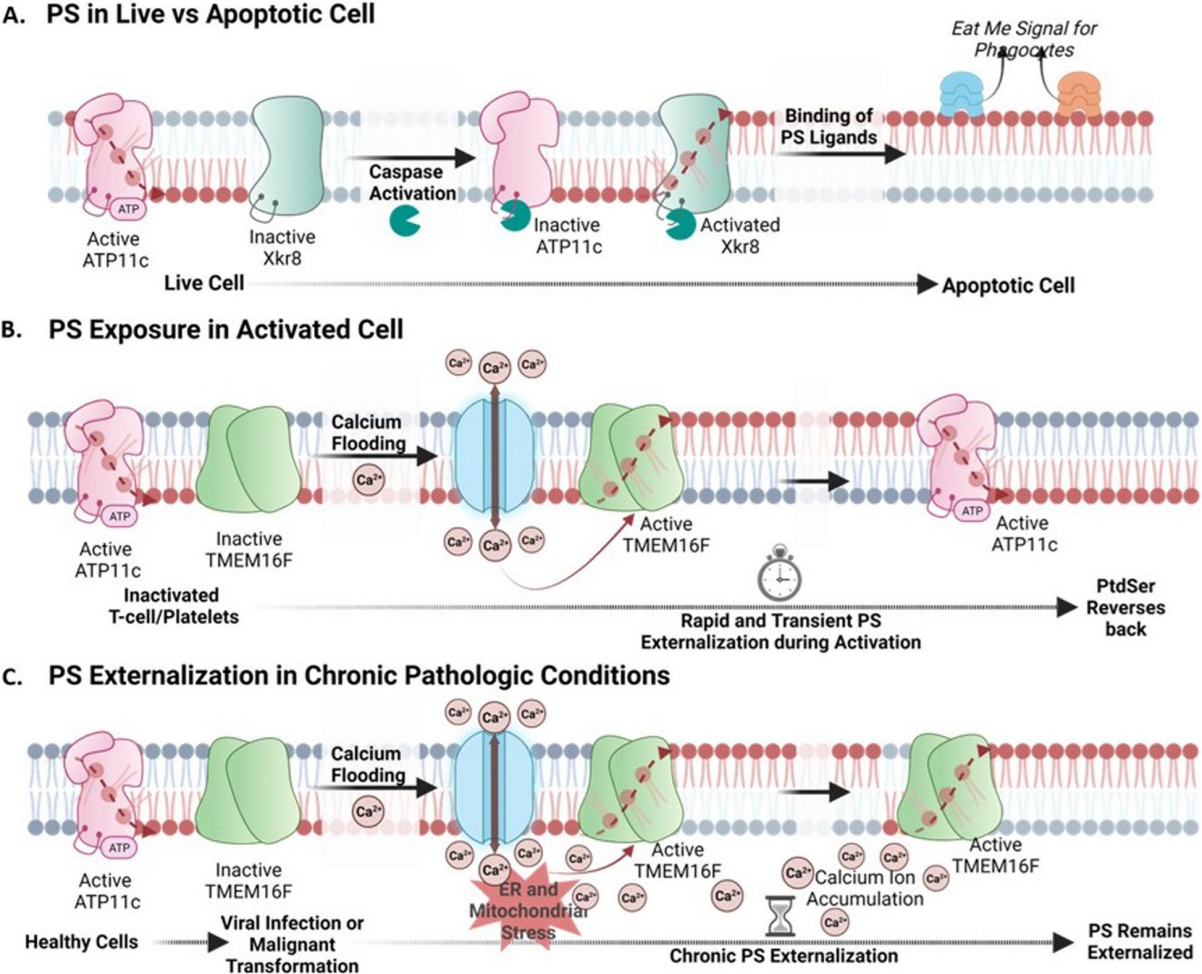
Chronic and reversible PS externalization by scramblases. (A) In live cells, PS is internalized due to the activity of P4-ATPase ATP11C and the inactivity of scramblase Xkr8. During apoptosis, caspases cleave ATP11C and Xkr8 at their caspase cleavage site, rendering them inactive and activated respectively. This leads to the irreversible externalization of PS and the binding of PS ligands, resulting in “eat-me signals” for phagocytosis. (B) Inactivated T cells and platelets internalize PS due to active ATP11C and inactive TMEM16F. Upon calcium influx into the cytosol (activation), TMEM16F forms an active dimer and externalizes PS rapidly and temporarily. Active ATP11C can then internalize PS again upon restoration of calcium homeostasis. Healthy cells typically possess active ATP11C and inactive TMEM16F, maintaining PS on the intracellular leaflet (C). Upon pathophysiological conditions of viral infection or malignant progression, calcium levels in the cytosol become constitutively elevated leading to constitutive TMEM16F activation and chronic PS externalization

Over the past decade, the regulated externalization of PS during apoptosis by caspase activation/proteolysis has been well characterized and the subject of many excellent reviews. Elegant studies championed by Shigekazu Nagata and colleagues have demonstrated that both the ATP11A and ATP11C PS flippases, as well as the Xkr8 (CED8) and Xkr4 PS scramblases, have activity-dependent caspase proteolytic cleavage sites that promote PS externalization during apoptotic cell death [25–29]. In this manner, by simultaneously (i) activating PS scramblases and (ii) inactivating PS flippases via irreversible proteolytic cleavage events, PS is also irreversibly and decisively externalized during apoptotic cell death where it acts as an emblematic signal for both caspase-dependent apoptosis and the subsequent efferocytosis (Fig. 3A, B). The biology of PS P4 ATP flippases, scramblases, and floppases is discussed in the following sections.

### Role of flippases, scramblases, and floppases in the distribution of PS membrane sidedness

At the molecular level, the (asymmetric) distribution of PS is dynamically regulated by at least three distinct types of lipid transfer enzymes that include (i) flippases, enzymes that catalyze ATP-dependent transfer of PS towards the cytosolic membrane, (ii) scramblases (most notably Xkr8 and TMEM16F) that randomly and passively scramble PS between the two leaflets in an ATP-independent manner, and (iii) floppases, enzymes that catalyze ATP-dependent transfer away from the cytosol [25, 24, 30]. Of these types, while important advances have been reported with flippases and scramblases, less is currently known molecularly and mechanistically about the floppases, such as ABC transporters, in part from the lack of assays to study floppase activity [25, 31]. Homeostatically, under resting conditions in viable cells, the maintenance of PS to the inner plasma membrane leaflet is primarily regulated by flippases, a subfamily of P4 type ATPases that form an acid-stable aspartyl phosphate with the phospholipid as it vectorially translocates lipids from the external leaflet to the cytosolic leaflet [32, 33]. In the past several years, several high-resolution X-ray crystallography and cryo-EM structures of flippases and scramblases with lipid substrates have been reported, and the mechanisms of substrate transfer and PS translocation have become much better understood [34–36].

For P4-ATPases, X-ray crystallography and cryogenic EM structures have been reported for several of the family members, ranging from budding yeast, fungi, and mammalian enzymes, including many of the conformation states of ATP-dependent catalytic translocation steps during phospholipid translocation [34, 37]. Indeed, the structural features are highly conserved between species and between family members, whereby the catalytic subunits have 10 trans-membrane spanning domains interspersed by a large cytosolic loop that forms the nucleotide-binding domain required for catalysis and flippase activity. This arrangement produces a tight channel for a single phospholipid with a hydrophobic gate in the middle that facilitates transfer that, in part, controls stoichiometry. In many P4-ATPases, the carboxyl-terminal domain acts as an inhibitor domain or plug that blocks and restrains flippase activity [33]. In addition, most P4-ATPases exist in the heterodimeric complex with an accessory beta-subunit from the ubiquitously expressed TMEM30 family, comprised of CDC50A, CDC50B, or CDC50C [38, 39]. As such, P4 ATPase except for ATP9A and ATP9B form a heterodimer with CDC50A, and CDC50 has been proposed to be a β-subunit of the flippase complex [40]. With the elucidation of these structures, the entry and exit gates have been described that involve a narrow groove that appears to exclude protein piggybacking and trafficking of protein cargo through the bilayer. Both ATP11A and ATP11C have two caspase cleavage sites near the P and N domains which bind nucleotides. When these sites are proteolytically cleaved, the flippase activity of ATP11A and ATP11C is catalytically inhibited [23, 29]. As a result, during caspase activation and apoptosis, PS remains externalized and fixed to the extracellular leaflet and serves as an eat-me signal for efferocytosis. While some recent studies indicate cell type specificity of P4 ATPase expression in tissues and cells [41], including data obtainable on the protein atlas [42], more research is needed in the area, particularly in cancer cells undergoing oncogenic and mutational evolution.

With respect to lipid scramblases, high-resolution structures have also been reported for both the caspase-activated (Xkr8) and calcium-activated (TMEM16F) subtypes as well as a recently described novel scramblase TMEM63b that is regulated by mechanotransduction and/or membrane structural thinning [43, 44]. In the case for the tertiary structure of the human Xkr8-Basigin scramblase complex (a caspase-activated scramblase), the high-resolution X-ray crystallography and cryogenic-EM structure reveals a stretch of amino acids that provide a membrane-spanning cuboid-like pore structure that defines the transmembrane pore region and mechanisms of lipid scrambling [45]. These structures and models reveal several important features at atomic resolution, including the association with its accessory protein Basigin/Neuroplastin [46], spatial parameters that define again the transfer akin to a credit card swipe mechanism whereby charged molecules in the transmembrane helix carry the phospholipids with insufficient room for protein piggybacking, as well as molecular details that define how caspase cleavage activates the channel [45]. Interestingly, these structures have also conceptually defined and rationalized “gain of function” point mutations (for example, W45A substitutions in Xkr8) that cause constitutive scrambling activity that results in PS externalization. Analogous to phospholipid flippases, the C-terminal inhibitory region acts as a plug [47]and that can be activated either by caspases or by sequential serine/threonine phosphorylation events [48]. A similar architecture of Xkr8 has been recently reported for Xkr9, revealing membrane-spanning domains that comprise the pore as well as a caspase-sensitive carboxy-terminal inhibitor domain located on the intracellular side [49], adding validation to aforementioned general features described for Xkr8. Both structures defined a C-terminal regulatory domain that functions to inhibit scrambling activity as a molecular basis of C-terminal cleavage and activation by caspases.

Although not involved in PS externalization for efferocytosis, the high-resolution X-ray and cryogenic-EM structures of TMEM16 (and related scramblases), which functions as a dual-function lipid scramblase and non-selective ion channel, have added additional mechanistic insight into this research field with respect to how lipids are transferred. The tri-dimensional structure of TMEM16F reveals a common homodimeric architecture where each protomer is composed of 10 transmembrane helices that can adopt multiple conformations to allow lipid translocation across the bilayer [50, 51]. During calcium-mediated activation, lipid transfer across the bilayer has been proposed again by a credit-card swipe model where the lipid polar headgroups penetrate and transverse the hydrophilic cavity while the hydrophobic tails remain anchored in the bilayer [52, 53], as well as a second calcium activation model whereby lipids thin the membrane enough to allow shuffling and scrambling [54]. In both models and analogous to Xkr8 and Xkr9, the lipid pores appear relatively restricted in diameter, allowing for the transport of lipids but not additional piggy-backed protein cargo.

Taken together, and despite differences in the mechanisms of phospholipid transfer, the emerging ideas from the tri-dimensional structures of both flippases and scramblases point to some commonalities in the mode of transfer and its implications in diseases, including cancer. First, most of the structures support a credit card swipe model of lipid transfer, where the channel/pore structure excludes protein piggybacking cargo, thereby ensuring the intracellular and extracellular PS binding proteomes are not interchanged (Fig. 1). Further studies should better explore (i) if/how PS bound to lipids sheds protein cargo during transfer, (ii) if PS molecules destined for transfer are selected free of proteins (for example if PS is externalized from a pool of de novo synthesized PS), or (iii) if some vectoral transfer of proteins is coupled with endocytosis or another mode of protein transfer. Second, in many of the structures, it has been observed that point mutations can lead to constitutive activation of scramblases or inactivation of flippases that predict constitutive or higher steady-state of PS on the extracellular surface (so-called PS-out cell phenotypes). In recent years, an increasing number of familial and somatic mutations have been identified in certain P4-ATPases and TMEM30 subunits, causing a range of congenital human diseases, including mental retardation, intrahepatic cholestasis, hearing loss, and red cell hemolytic anemia [55]. In cancer cells, sporadic mutations in TMEM30A have been linked to lymphomas such as diffuse large B-cell lymphoma (DLBCL) [56] and follicular lymphoma [57], although it still remains to be determined if the oncogenic potentials are directly related to PS externalization. Recently, studies employing genetic ablation of TMEM30A in leukemia cells render these cells constitutively PS positive and resistant to NK-mediated degranulation and cell killing, suggesting that PS externalization may serve as an immune-evasive strategy in malignancies [58]. As discussed again below, the recent characterization of somatic mutations in P4-ATPases, TMEM30A proteins, and PS scramblases could implicate a broader involvement in solid cancers that may contribute to PS dysregulation in tumors and potentially a mechanism for cell intrinsic immune escape in cancer via constitutive PS externalization observed in many solid cancers [59].

### Externalization of PS changes the PS proteomes from an intracellular homeostatic PS itinerary to an extracellular PS itinerary

As a result of irreversible PS externalization during cell death, an emblematic signature for apoptosis, dramatic changes in the localizations of the PS proteome(s) thereby occur, whereby the depletion of PS on the inner membrane compromises the above-mentioned targeting of intracellular signaling proteins (the homeostatic proteome), and at the same time serving to recruit a large and diverse class of PS-binding proteins on the extracellular side of the membrane (the extracellular proteome) that can promote efferocytosis (Fig. 1). Such PS binding proteins include important bridging molecules such as MFG-E8/Del-1 and Gas6/Pros1 that indirectly link integrins (αvβ5 and αvβ3) [60, 61] and TAM (Tyro-3, Axl, and Mertk) receptors [62–64] respectively, as well as many other receptors such as LOX-1 [65, 66], CD300 [67, 68], RAGE [69, 70], Stabilin-2 [71–73], (T-Cell/Transmembrane, Immunoglobulin, and Mucin) TIM family (TIM-1, TIM-3, and TIM-4) [74–77], uPAR [78], BAI-1 [79], C1rR/scavenger receptors [80] and others that directly interact with externalized PS (Fig. 1). In the case of MFG-E8 and Del-1, which contain C1 and C2 PS binding domains [60, 81], and Gas6 and Pros1, which contain vitamin- K dependent γ-carboxylated PS binding domains [82–84], these proteins directly enter the secretory pathway and excluded from binding and being trapped by intracellular pools of PS generated by PS-S1 or PS-S2 (Fig. 2). Additionally, for Gas6 and Pros1, enzymatic γ-carboxylation of the Gla domain occurs in the lumen of the ER [82], notably partitioning it from intracellular pools of PS. While the consequences of the outward flow of PS on efferocytosis are clear, the consequences from the depletion of the intracellular PS pool are only beginning to be appreciated. For example, recent studies have shown that PS externalization inhibits intracellular Src activity (pY416) and its phosphorylation of downstream targets, as well as changes the anchoring of the actin cytoskeleton [85]. PS externalization has also been linked to increased cytosolic accumulation of Annexins, such as Annexin A1 and Annexin A2. Whether the loss of the PS binding protein itinerary contributes to the efferocytosis process by altering membrane shape or blebbing is an intriguing but not well-understood possibility.

The PS externalization that occurs during apoptosis and the engagement of PS receptors and efferocytosis is one of the best-understood functions for externalized PS. Nonetheless, the wide array of PS binding proteins and PS receptors that contribute to efferocytosis suggests redundancy and clearly highlights the complexity of this biology. Several models suggest that PS receptors can act cooperatively or binary. For example, αvβ5 integrin [86], αvβ3 integrin, or TIM-4 [87] can all cooperate with Mertk, to act synergistically in a model called “tickling and tethering”. In this model, one of the PS receptors anchors the apoptotic cell via exposed PS and the second receptor stimulates cytoskeletal organization and efferocytosis, typically via Rac1 activation and the alteration in the actin cytoskeleton [86, 88]. However, while not yet experimentally defined, other studies posit more elaborate PS-binding receptor proteomes, akin to a phagocytic or efferocytic synapse, whereby multiple receptors cluster to promote actin-mediated efferocytosis [89, 90] In some cases, cell-type specific pairs mediate efferocytosis, such as the use of αvβ3 integrin and MFG-E8 in tangible-body macrophages [61] and the role of Axl on alveolar macrophages [91, 92]. Notably, however, in some cases, such as bone marrow derived macrophages, in several tissue resident macrophage subsets, or in M2c and M2d tumor-associated macrophages, single knockout of PS receptors, most prominently Mertk, can compromise efferocytosis [93–95]. This predicts that vulnerabilities can be exploited despite redundancy to target PS and efferocytosis as a therapeutic modality.

### PS externalization on viable cells is distinct from caspase-mediated PS externalization on apoptotic cells

In contrast to the above-mentioned events associated with apoptosis and subsequent efferocytosis, PS is also transiently externalized in living cells under a variety of important physiological cell activation events, including during T cell receptor stimulation [96, 97], B cell receptor stimulation [98, 99], thrombin-induced platelet and endothelial activation [100–102], and when cells are stimulated with certain growth factors such as PDGF and VEGF that transiently regulate PI3-kinase which, in turn, transiently mobilizes intracellular calcium. PS is also externalized in myoblasts and trophoblasts for physiological cell fusion events associated with myotube formation [103, 104] and trophoblast fusion and placenta development [105, 106]. More recently, it has been shown that PS can be externalized on damaged cells as a mechanism for pore-induced membrane repair, where PS externalization induces membrane plasticity and repair [107], and in some cell types, PS externalization leads to the formation of extracellular vesicles or exosomes [108–110].

### PS externalization by intracellular calcium signaling

A common theme in many of the aforementioned scenarios that involve transient cell activation on viable cells is that the reversible PS externalization appears to be regulated by increased intracellular calcium concentrations. Elevated intracellular calcium serves to subsequently activate members of the calcium-regulated lipid scramblase TMEM16 family members, a diverse family of multi-membrane ion channels and membrane-spanning lipid scramblase proteins that passively scramble lipids (including PS) in an ATP-independent manner across the lipid bilayer [52] (Fig. 3C). In the case of calcium-mediated PS externalization on platelets, mechanistic studies show that the rises in cytosolic calcium which control most platelet responses in thrombosis and hemostasis are regulated by the ORAI1-STIM1 pathway [111], which may be common to other cell types, including endothelial cells and cancer cells. In platelets, in response to the stimulation of the ITAM-linked collagen receptor, glycoprotein VI (GPVI) and treatment with the endoplasmic reticulum ATPase inhibitor, thapsigargin, calcium entry fully relies on ORAI1-STIM1 pathway and is strongly suppressed by protein kinase C (PKC) activation. Subsequently, upon the transient intracellular calcium, TMEM16F transiently externalizes PS in a tightly regulated calcium-dependent manner, such that once intracellular calcium returns to baseline, cellular flippases return PS to the inner leaflet of the plasma membrane. In terms of the relevance of TMEM16F to the thrombin-activated platelets, hereditary mutations in TMEM16F have been identified in patients with Scott syndrome, a rare bleeding disorder whereby coagulation is impaired by the lack of calcium-mediated PS externalization on the platelet membranes [102, 112].

**Fig. 4:**
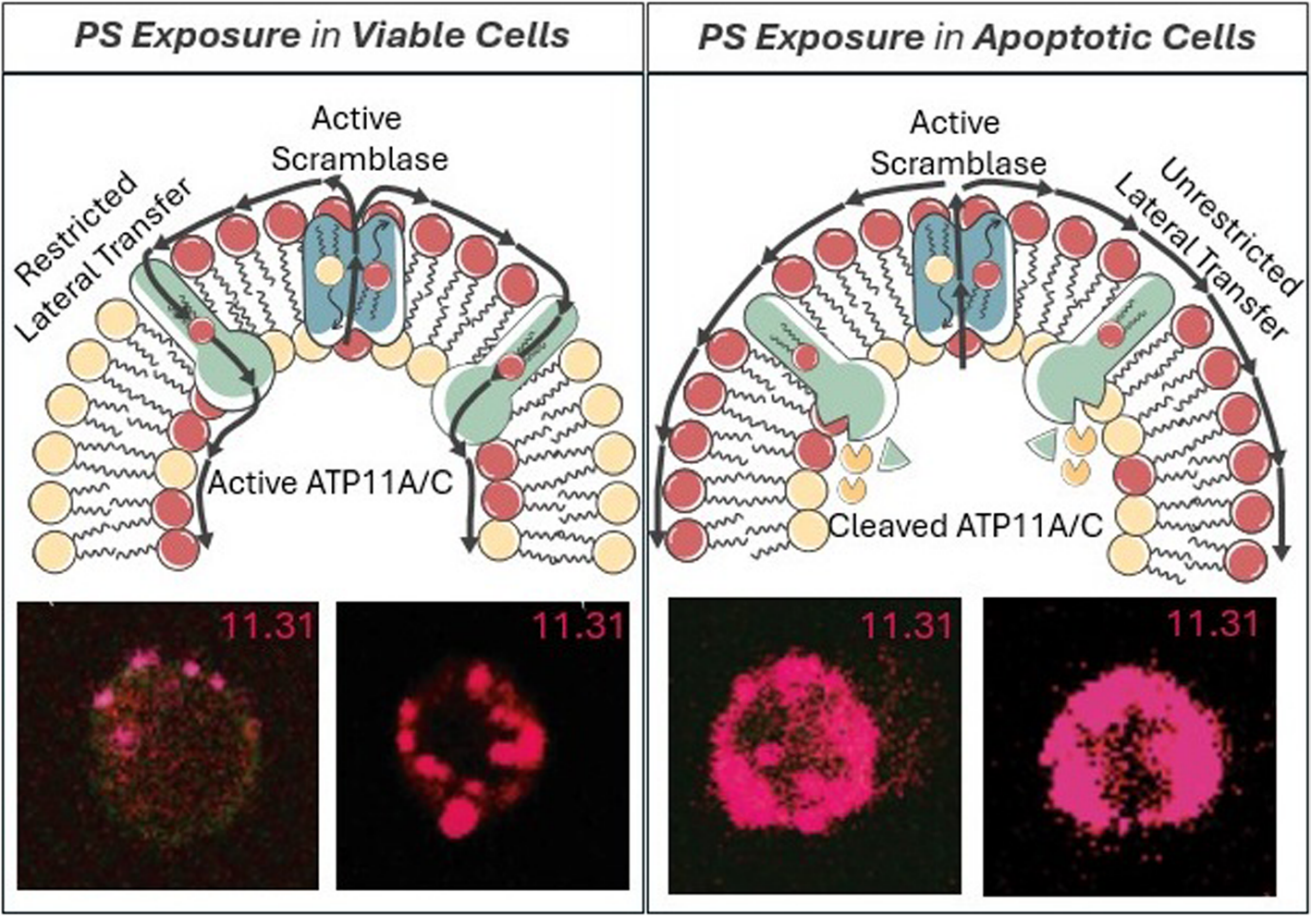
PS externalization in live versus apoptotic cells. Viable cells (left) have PS externalized (shown by the binding of PS-targeting antibody, 11.31) by scramblase TMEM16F in a punctate, localized manner. This is then brought back in due to the hydrolysis of ATP to ADP and the resulting activities of ATP11C. In contrast, PS exposed on apoptotic cells (right) due to caspase cleavage of ATP11C and scramblase Xkr8 show PS externalized throughout the entirety of the PM in a less localized and more uniform manner [115]

At the biochemical level, it is important to note that PS externalization on activated viable cells is phenotypically distinct from the PS externalized on apoptotic cells, as it is rare to observe PS-positive live activated immune cells, cancer cells, or platelets efferocytosed. Several factors likely contribute to these differences. First, live activated cells retain and express don’t-eat-me signals such as CD47 and CD31 that function to resist efferocytosis [113]. Moreover, in contrast to the caspase-mediated inactivation of flippases described above during apoptosis, live activated cells retain functional flippase activity to reversibly internalize PS to re-establish intracellular PS interactomes [29]. As a result, on viable cells, but not on apoptotic cells, any lateral transfer of PS following scrambling and externalization would be restricted by rapid and efficient internalization of PS through the functional ATP-dependent flippase activity to re-establish PS asymmetry and prevent robust externalization of PS on viable cells (Fig. 4). It is possible that flippases might “react” to the PS externalization by scramblases, either via co-localization with active scramblases or some crosstalk occurs between scramblases and flippases to alter the kinetics of flippase activity when scramblases become activated. Studies by Herrmann and colleagues showing that apoptotic cells, but not viable PS-positive cells, exhibit cooperative binding kinetics to Annexins would support this idea [114]. By employing labeled PS-targeting monoclonal antibodies Bavituximab and 11.31, the observed patterns of PS externalized on apoptotic T cells versus activated T cells stained by PS-targeting mAbs show distinct staining patterns [115], whereby PS externalization during apoptosis results in high surface density/intensity of irreversibly externalized PS that leads to efferocytosis, while the PS externalized on CD3/CD28 TCR activated T cells is punctate and focally distributed that resemble ports of exits (Fig. 4, lower panels) [115]. Such lateralization of PS might also be a driving force for the clustering of PS receptors, since some PS receptors, mainly TAMs, require dimerization of oligomerization for activation [83, 116]. The combination of the (i) reversible PS externalization, (ii) the retention of don’t eat-me signals, and (iii) functional ATP-dependent flippases all likely contribute to the differences in function of externalized PS on the surface of viable versus apoptotic cells.

While the externalization of PS on viable activated cells does not appear to be sufficient to induce efferocytosis, it is clear that cell activation (for example, during T cell activation or platelet activation) also results in rapid and dynamic changes to the PS proteomes, whereby intracellular cytosolic proteins that typically rely on PS to localize to the inner side of the cytosolic membrane lose membrane attraction to be rendered cytosolic, and PS on the outer plasma membrane surface recruits a new extracellular proteome. Indeed, with respect to the outward flow of PS externalization on live activated cells that does not result in efferocytosis, PS is involved in several biochemical and physiological processes that include, but are not limited to, (i) the activation of PS-dependent extracellular enzymatic catalytic events, (ii) the recruiting and encrypting of coagulation factors to induce fibrin and thrombin for clotting, and (iii) the recruitment of local immune resolution factors that dampen inflammation and promote immune resolution to reestablish and resume homeostasis. With respect to enzymatic catalysis, examples where PS is involved in extracellular catalysis include the activation of extracellular membrane-bound sheddases, such as ADAM17 and ADAM10-mediated ectodomain cleavage [117, 118], as well as in the activation of membrane-bound extracellular protein-disulfide isomerases (ePDI) [119]. In the case of ePDI activity, which involves the rearrangement of thiol/disulfide bonds and the resulting allosteric changes in the conformations of extracellular receptors and cargo, recent studies suggest that protein disulfide isomerase activities are involved in both PS externalization, as well as the resulting binding of several Gla-containing coagulation factors to PS. In platelets, inhibitors of protein disulfide isomerase activities can block exposure of PS, while conversely, certain ePDIs can increase PS exposure on activated platelets [120, 121]. More recent studies have shown that TMX4, a membrane-type ePDI family, when depleted in platelets or endothelial cells by conditional knockout, inhibits both αIIb/β3 activation and platelet aggregation as well as PS externalization; these events could be reversed by recombinant TMX4 [122]. Presently, it remains unclear if comparable cysteines in P4-type flippases are also functionally labile to ePDI activity and, if so, what ePDIs catalyze such thiol-disulfide isomerization reactions.

In addition to the externalization of PS by ePDI activity on activated platelets, the binding of several of the Gla domains to PS is also dependent on ePDI activity [119]. For example, PS externalization on calcium-activated platelets and calcium-activated endothelial cells recruit coagulation factors II, VII, IX and X and activated factor VII (FVIIA), Tissue factor (FX) and Factor X via their γ-carboxylated Gla domains to establish the coagulation initiation complex upstream of thrombus formation. In these studies, it was shown that membrane impermeant thiol blockers or PDI inhibitors, added after platelet stimulation and after phosphatidylserine exposure to exclude their influence on primary platelet activation, significantly inhibited binding of coagulation factors to thrombin-stimulated platelets. Since the Gla domains of all coagulation factors have conserved cysteines, it appears ePDI mediated thiol-disulfide isomerization may represent a generalized activation strategy for platelet activation. Whether other Gla-containing PS-binding proteins, such as Gas6 and Pros1, require ePDI should be explored. However, not all PS externalization in activated viable cells is driven by elevation in intracellular calcium. For example, during inflammation and the release of cellular ATP, ATP can induce PS externalization in a TMEM16F-independent manner, via the binding to the P2X7 receptor and plasma membrane complex involving P2X7 and the ER chaperone protein EROS to induce PS scrambling and externalization [123].

### Immune modulation of externalized PS I: Signaling on viable cells by transient PS externalization

**Fig. 5:**
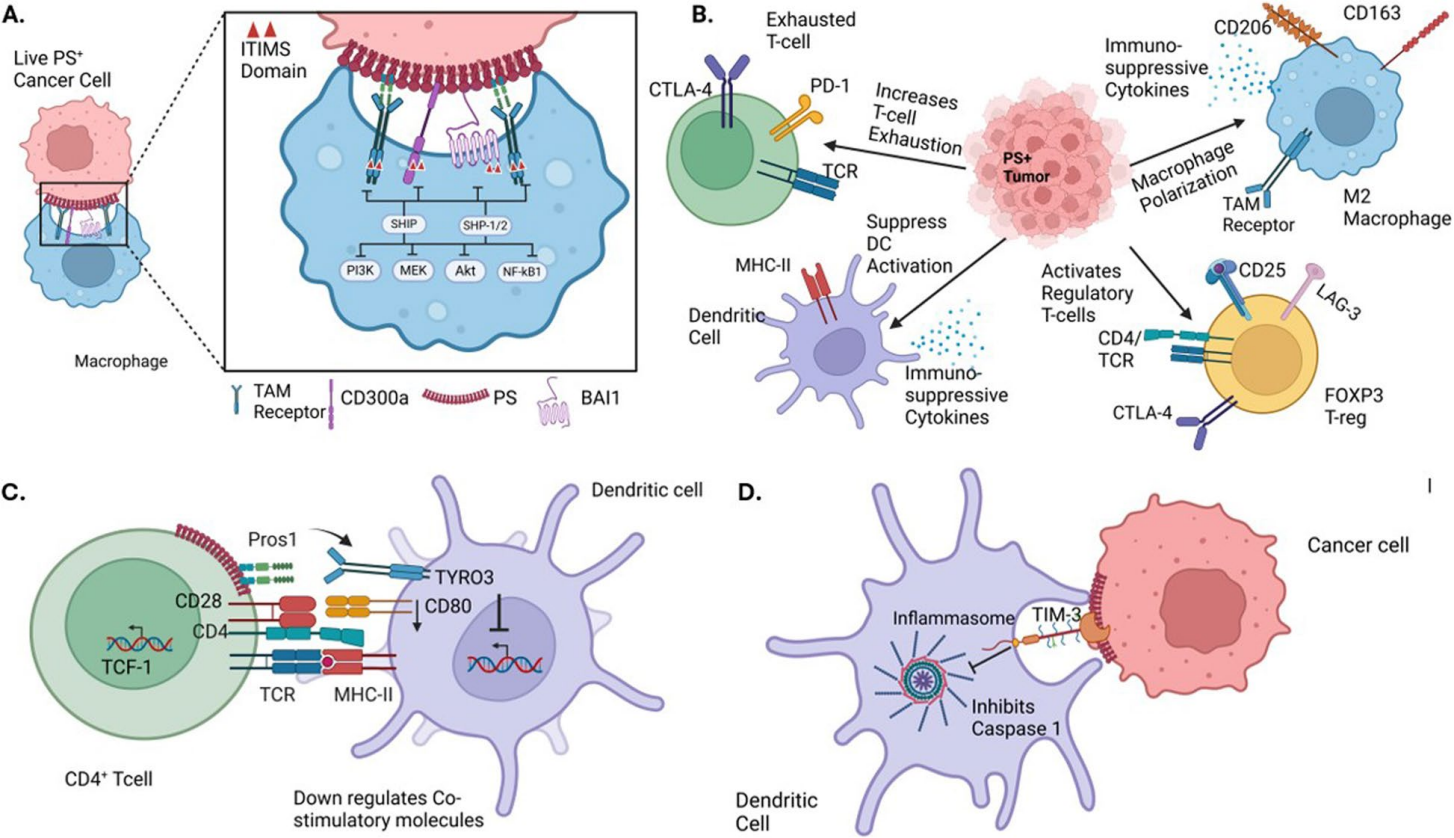
Immunomodulatory effects of PS externalization in the TME. A. Live PS-expressing cancer cells engage PS-binding TAM, CD300a, and BAI1 receptors on macrophages. These receptors contain ITIM or ITSM motifs, which recruit SHP1/2 or SHIP phosphatases to inhibit downstream pro-inflammatory signaling pathways, including NF-κB, Akt, MEK, and PI3K, promoting immune suppression. B. Chronic PS exposure in the TME sustains immune suppression by polarizing macrophages to M2 macrophages, converting DCs toward resolving phenotype, increasing T cell exhaustion, and activating Regulatory T cells. C. Activated T cells secrete Pros1, a ligand for Tyro3 expressed on dendritic cells, dampening antigen presentation and limiting co-stimulatory molecule expression. D. PS externalization on cancer cells can also inhibit the NLRP3 inflammasome via TIM-3 receptor engagement, dampening inflammatory signaling (see text for details)

Beyond the above-mentioned distinctions in the mechanisms of PS externalization on live activated cells versus apoptotic cells in terms of their requirements for PS scramblases and distinct surface localization patterns, the immunological consequences of PS externalization on viable versus apoptotic cells also appears to be distinct. Externalized PS on viable cells can participate in both cell extrinsic and cell intrinsic modes of immunomodulation. For the cell extrinsic effects, where PS externalization on viable activated cells suppresses neighboring cells via a juxtacrine mechanism, PS externalization has been shown to engage immunoreceptor tyrosine-based inhibitory motifs (ITIM) receptors on immune subsets to dampen inflammatory pathways such as the inhibition of NF-κB, Toll-like receptors (TLR’s) and NLRP3 inflammasomes. As noted in Fig. 5A, several of the PS receptors that engage externalized PS, including Mertk, Tyro3, Axl, BAI1, and CD300b have immune-inhibitory ITIM or ITSM motifs that when tyrosine phosphorylated bind to the SH2 domains of SHP1 or SHIP phosphatases to negatively regulate post-receptor signaling (Fig. 5A, SFig 1A). In the recent studies by Xu and colleagues, these authors have described molecular signatures that define and compare SHP1, SHP2 and SHIP binding sites to inhibitory receptors [124]. Accordingly, certain PS receptors such as CD300a have bivalent motifs that would favor docking to SHP1/SHP2 whereas Mertk and other TAM receptors have split ITIMs that would favor docking to lipid phosphatases (Fig. 5A, SFig 1), and in doing so, influence multiple cell types including T cell exhaustion, suppression of DCs, M2 macrophage polarization, and induction of T regulatory cells (Fig. 5B).

In addition to the cell-to-cell negative regulation, activated cells can also employ local paracrine and autocrine signals using PS as dampening signals. An interesting example of this mode of regulation occurs following T cell activation, whereby stimulated cells up-regulate and secrete Pros1, a Tyro3 and Mertk ligand that can both negatively regulate T cells in cis (autocrine) [125] but also feedback to suppress antigen-presenting cells and antigen presentation and limit the magnitude of DC activation [126]. In this latter model, a conditional knockout of Pros1 in T cells leads to an increase in co-stimulatory molecules and enhanced immune responses to T cell-dependent antigens [126], ultimately controlling the magnitude and duration of T cell activation and type 2 responses [127] (Fig. 5C). Functionally, PS inhibitory signals are important to return T cell activation to baseline to prevent overactivation of immune responses and cytokine storm. Moreover, on both cancer cells and T cells, up-regulated PS can feedback and inhibit TIM3 and subsequently inhibit the inflammasome to ultimately affect MHC activation and T cell activation [128, 129] (Fig. 5D). Modulation of TIM3 is critical to maintain homeostasis and dampen inflammation and over activation of the immune system. As noted below, these inhibitory effects become commonly chronic and sustained in the tumor microenvironment leading to impaired host immunity.

### Immune modulation of externalized PS II: Signaling on apoptotic cells/efferocytosis by fixed PS externalization

**Fig. 6:**
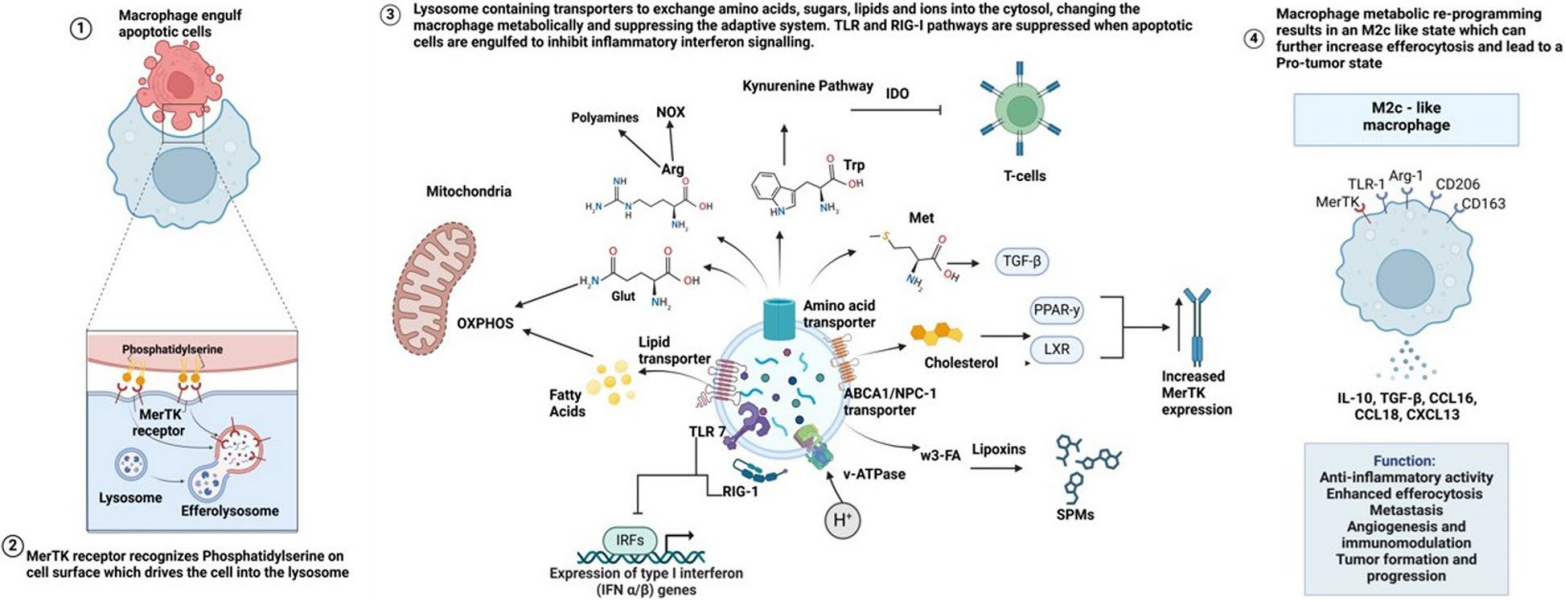
Efferocytosis of apoptotic cells, lysosomal degradation, and efferocyte reprograming. (Panel 1) Externalized PS is an emblematic signal for both recognition and trafficking of apoptotic cells to the phagolysosome. (Panel 2) Mertk is proposed to act as a “trojan horse” to traffic apoptotic cells to intracellular itineraries and the lysosome. (Panel 3) Phagolysosomes then degrade and recycle components of the phagolysosome to the cytosol and other intracellular compartments for phenotypic reprogramming. (Panel 4) Macrophage phenotype is now an M2c-like state wherein efferocytosis can be furthered and a pro-tumoral environment can be achieved. These M2c macrophages are thought to promote tumor progression and metastasis while suppressing inflammation

In contrast to the cell-to-cell contact and suppression/resolution of inflammation by PS externalization in live activated cells, PS externalization on apoptotic cells promotes efferocytosis. In the case of professional macrophages, which can engulf multiple apoptotic cells, and, in doing so, conceivably double, triple, or quadruple their mass, digestion of cargo in phagolysosomes results in transport of amino acids, lipids, cholesterol, and nucleotides to the cytosol that have profound reprogramming effects on the efferocyte that can influence metabolism, transcription, and translation [130–132]. With respect to the trafficking and digestion of apoptotic cargo, several PS receptors can facilitate and direct trafficking of cargo to an intracellular itinerary akin to a trojan horse model of delivery. For Mertk, which binds to PS indirectly via Gas6, Merk has been shown to facilitate phagolysosomal trafficking for apoptotic cells (Fig. 6 panels 1 and 2) and rod outer segments [133], as well as during LC3-mediated phagocytosis [134]. However, unlike during the stoichiometric turnover of cellular macromolecules during autophagy, whereby there is no net increase in cargo, phagolysosomes digesting apoptotic cells must adapt to anabolic nutrient conditions from increasing their mass. Moreover, unlike autophagy, cells undergoing efferocytosis must also survey the extracellular cargo, analogous to a type of molecular autopsy. In recent years, efferocytosis followed by phagolysosome maturation and nutrient cargo recycling has gained considerable attention and implicated in many important aspects of metabolism and immune regulation [131].

In the case of cholesterol, which is poorly metabolized after recycling from the phagolysosome in the phagocytic cell, cytoplasmic cholesterol particles can act both in a homeostatic role and a pathophysiological function during atherosclerosis. Under homeostatic conditions, cholesterol efflux from phagolysosomes and increased cellular cholesterol targets nuclear receptors such as PPAR-γ and LXR/RXR to transcriptionally upregulate anti-inflammatory factors as well as Mertk receptors [135]; in the latter case, Mertk acts in a feed-forward mechanism to further drive additional efferocytosis and expand on the anti-inflammatory and resolving functions of efferocytosis. In addition to activating transcriptional pathways, cholesterol also interacts with ABCA1, a key mediator for intracellular cholesterol efflux to Apolipoprotein A-1 (ApoA-I) for the safe generation of high-density lipoprotein (HDL), a complex also involved in anti-inflammatory responses [136, 137]. Other lipids, such as omega-3 fatty acids and long chain fatty acids when recycled can be converted into specialized pro-resolving mediators (SPMs) by lipoxygenases that produce lipoxins and resolvins as generalized negative immune regulators [138].

Another emerging and important aspect of phagolysosomal recycling involved the rapid flooding of amino acids via engagement of amino acid transporters in the phagolysosomal membrane (Fig. 6). Several potent immunomodulatory functions of amino acids have been reported over the past several years, including general amino acid sensing pathways involving the mTOR/S6 kinase pathway. Moreover, as expanded below, several of the amino acid transporters, involving amino transporters such as the transport of tryptophan, the transport of arginine, the transport of methionine, and the transport of glutamine contribute to both anti-inflammation and the re-polarization of macrophages towards M2, and/or the maintenance of an immature DC phenotype (Fig. 6, summarized in panel 3). In the case of tryptophan, recent studies by Sukka et al*.* have elegantly shown that efferocytosis is linked to tryptophan metabolism by IDO1 and IDO2 to kynurenine [139], an immunosuppressive factor that drives IL-10 and, ultimately, the generation of T regulatory cells. Additionally, methionine flooding in macrophages has been linked to the production of S-adenylmethionine (SAM), a methyl donor to DNA methyltransferase 3A (DNMT3A), and for the ultimate production of TGF-β [140], while arginine has been linked to putrescine and anti-inflammatory polyamines, such as the inhibition of IL-1β and IL-6 [141]. Finally, glutamine flooding is associated with glutaminase and glutamate dehydrogenase as well as the production of α-ketoglutarate which fuels the TCA cycle [142]. Together with the transport of fatty acids for beta oxidation, the transport of glutamine appears to be one of the main drivers for the shift in efferocytosing macrophages from glycolysis to oxidative phosphorylation, and the shift from M1 to M2 phenotypes (Fig. 6, panel 4) [131]. Similar types of reprograming phagocytosing efferocytes have been reported in DCs, leading to down-regulation of antigen-presentation and suppression of inflammation [143, 144].

In some cases, the activation of amino acid or lipid transporters itself can be coupled with suppression of TLR and NOD-Like receptors that inhibit inflammation. Indeed, recent studies indicate that endolysosomal amino acid transporters (EL-aa) possess several unique functions, including their ability to assemble large scaffolds to recruit signaling complexes. For example, the solute carrier family 15 member 4 protein (SLC15A4) transporter has been shown to recruit NOD1, TLR7, TLR9, and AP3 complexes which can inhibit many cytokines and inflammatory mediators [130]. Similarly, SCL15A3, another member of the SLC15 family, recruits NOD1, RIPK2, to inhibit IL-6, IL-1β, and STING [145]. Moreover, amino acid and fatty acid flooding of cells following phagolysosomal transport can alter central metabolism and mitochondrial bioenergetics. For example, both amino acid and fatty acid flooding increase mitochondrial beta oxidation and decreases glycolysis. This ability to sense immunogenic cargo, in addition to the aforementioned metabolic changes, permits integration of metabolism and immune function.

In the case of the engulfment of apoptotic cells, efferocytic macrophages (Meff) are distinct from the classical anti-inflammatory (M2) phenotype due to their specialized role in efficiently clearing apoptotic cells. For example, efferocytosis reprograms macrophages to continually engulf and process apoptotic cells, enabling them to clear large amounts of cellular debris efficiently. This process relies on mechanisms like the Pol II pause/release for rapid transcriptional responses necessary for ongoing efferocytosis mentioned above [132]. Also, the induction of the SLC gene program enhances glucose uptake and fuels macrophage metabolism [146, 147]. Furthermore, efferocytic macrophages shift their metabolism toward fatty acid oxidation and mitochondrial respiration, utilizing apoptotic cell-derived fatty acids to produce energy and promote anti-inflammatory responses critical for tissue repair [148]. The metabolic shift is also linked with nuclear programs such as activating LXR and PPARδ nuclear factors, which coordinate lipid metabolism and further suppress inflammation [149]​. Indeed, recent studies suggest that efferocytosis is not uniform across all macrophages; it shows considerable heterogeneity, with different macrophage subsets [150]. Understanding this heterogeneity is crucial, especially in pathological states like cancer, where efferocytosis can contribute to tumor progression. To explore these differences, methods such as single-cell RNA sequencing and spatial transcriptomics will shed more light onto such novel macrophage subsets.

### Immune modulation of externalized PS III: Signaling on chronically diseases cells

The physiological regulation of PS externalization during apoptosis and cell activation and apoptosis are part of normal homeostatic mechanisms. Both mediate normal physiological functions in tissues necessary for recovery and homeostasis. However, the physiological processes that regulate PS biology can go awry in pathological conditions and contribute to chronic disease and tissue and immune dysfunction. Constitutive PS externalization has been observed in chronically stressed cells in both the tumor microenvironment and in virus infected tissues and represents one of the most agnostic and repetitive conditions in the context of chronic pathophysiology [59, 151]. Moreover, unlike protein targets that mutate with advanced disease progression and grade, which is compounded following conventional chemotherapies and targeted therapies, and the glycocalyx, which becomes vastly modified in cancers, externalized PS represents a stable epitope in tumors with no known or reported chemical modifications to the anionic head group. In the case of other non-protein targeting of the carbohydrate modifications, for example, as a result of glucose deprivation in the tumor microenvironment, both N and O-linked glycosylations shorten, such that like protein targets, tumor glycocalyx (sialic acid, fucose, N and O-linked glycosylations) are unstable that substantially change as tumors progress making them more difficult to target. Aberrant glycosylations in tumors also include abnormal branching of N-linked glycans (N-glycans), truncated O-linked chains, and diverse fucosylated and sialylated terminal structures. As a result, unlike proteins and glycans, since the structural integrity of PS remains relatively stable, PS-targeting therapeutics conceivably are predicted to have a vastly reduced likelihood of developing resistance. While therapeutic resistance to targeted therapies is a significant challenge in cancer treatment, allowing cancer cells to evolve and evade immune responses, this is expected to be minimized or spared with PS-targeting therapeutics.

In tumors, which are often referred to as “wounds that never heal” [152], many aggressive solid cancers exist in a state of high apoptotic indexes, characterized by high proliferative rates and high apoptosis rates. Such high apoptotic indexes in tumors necessitate PS-mediated efferocytosis by professional phagocytes such as DCs and macrophages that are notably linked to the secretion of suppressive cytokines such as IL-10 and TGF-β, and the complex events associated with efferocytosis noted above. Indeed, as Ucker and colleagues point out, clinical data has linked high apoptotic indexes with poor overall survival in solid cancers [153], providing a rationale for the targeting of PS receptors or the blockage of PS as an inhibitory modality, as further discussed below. Excessive efferocytosis in tumors also results in compensatory proliferation, VEGF production and neovascularization, coagulopathy, and the production of tolerogenic cytokines and chemokines that support complex pro-tumorigenic microenvironment.

The idea that high apoptotic indexes and PS externalization on apoptotic cells contribute as a cell intrinsic signal to immune evasion is supported by recent evidence that Xkr8 knockdown on cancer cells, which prevents PS externalization during caspase-mediated apoptosis, enhances chemotherapy efficacy and stimulates immune host anti-tumor immunity in both subcutaneous and orthotopic pancreatic solid tumor models [154]. Interestingly, in the aforementioned models, Xkr8 knockdown also enhanced the efficacy of certain chemotherapies such as 5FU and anthracyclines (oxoplatin) that have been linked to immunogenic cell death, a form of caspase-dependent cell death associated with the release of danger signals such as CRT, HMGB1, and ATP that override PS immunosuppressive signals and trigger DC cross-presentation and T cell activation [155, 156]. In the context of Xkr8 and PS externalization, it will be important to investigate if inhibition of Xkr8 can improve the benefits of immunogenic cell death induction or other types of non-apoptotic cell death such as necroptosis, pyroptosis, ferroptosis, and cuproptosis. For example, in the case of ferroptosis, which is accompanied by excessive lipid peroxidation and oxidation of PS, there is some controversy as to when this process is immunogenic or tolerogenic [157]. Also, many tumor microenvironments are heterogeneous whereby different forms of cell death can occur in different regions of the tumor. Hypoxic tumors with low ATP are more likely to experience regulated necrosis [158], which cells at the tumor margin, as the gateway cells to the tumor mass, are more likely to experience apoptosis and have anti-inflammatory features. Based on the results of the Xkr8 knockdowns that improve host tumor immunity, development of selective inhibitors of Xkr8, or characterization of relevant Xkr8 kinases, warrant further investigation. Xkr8 can also be activated by phosphorylation and hence it will be interesting to assess whether oncogenic kinase cascades also impinge on the Xkr8 scrambling pathway and PS externalization [48].

### Stress-induced PS externalization: Role of Calcium and TMEM16F

**Fig. 7:**
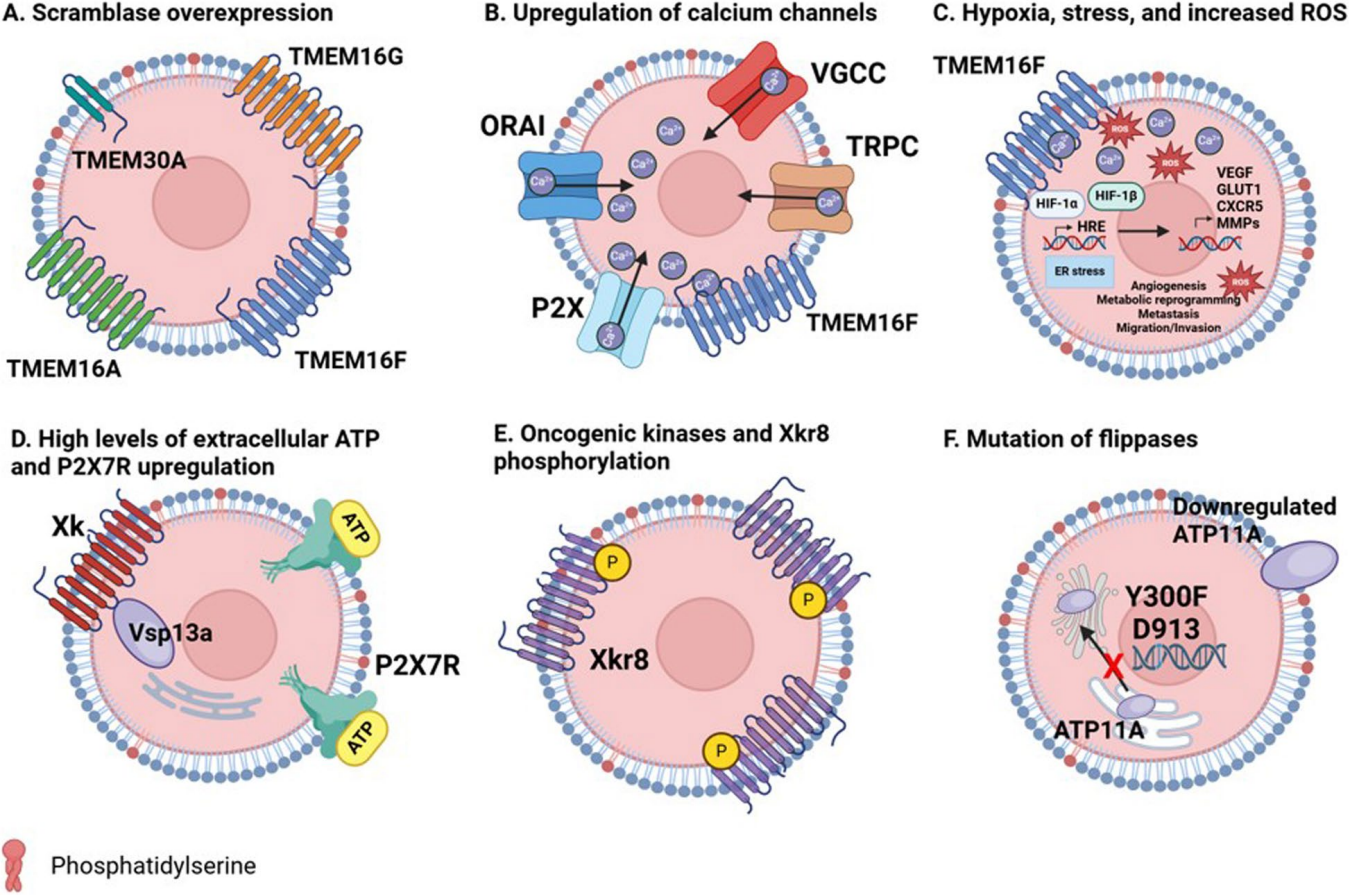
Mechanisms for chronic PS externalization in the TME. Several mechanisms have been proposed for the tonic sustained PS externalization in the tumor microenvironment that include scramblase overexpression (**A**), upregulation/elevated levels of calcium channels (**B**), hypoxia and ROS (**C**), increased extracellular ATP (**D**), oncogenic kinases phosphorylating Xkr8 (**E**), and mutation of flippases all contribute to the constant PS externalization in the TME that leads to a cold tumor phenotype (**F**)

While the high extracellular PS contributed by apoptotic cells from caspase activation and caspase-activated scramblases such as Xkr8 can be conceptually rationalized, the high extracellular PS contributed by dysregulated viable cells in the tumor microenvironment is likely equally important for driving inhibitory pathways and immune suppression, but much less well understood at the molecular and cellular level. As noted above, discovery of a calcium-activated PS scramblase activation platform clearly links cell stress and the ensuing PS externalization with a classic tumor signal related to the dysregulation of intracellular calcium and hypercalcemia (Fig. 7A. Indeed, TMEM16F, a PS scramblase, is activated by influx of calcium into the cytosol [102, 159, 160]. Moreover, in the tumor microenvironment, there is upregulation of multiple calcium channels, receptors, and pumps [161–164] (Fig. 7B). These include transient receptor potential channels (TRPVs) 4/6 and 7/8 [165, 166], stromal interaction molecules 1/2 (STIM1/2) [164, 167], purinergic receptor ion channel 7 (P2X7) [168], inositol trisphosphate receptor (IP3R) [169, 170], and other receptors and pumps [161, 163, 164, 171]. Such upregulation results in constant hypercalcemia within the tumor, potentially leading to the chronic activation of TMEM16F and thus the constitutive externalization of PS in the tumor microenvironment.

Another important aspect of the tumor microenvironment is chronic hypoxia (Fig. 7C). Chronic hypoxia has been linked to PS externalization on both cancer cells and tumor vascular endothelial cells and was an impetus for the original development of the PS targeting antibodies such as Bavituximab as discussed below. More recently, Contag and Hermiston have also reported PS externalization as a feature for cancer stem cells [172]. Like hypoxia, tumors also exist in metabolic stress, resulting in glucose and amino acid deprivations. Glucose deprivation can lead to impaired glycosylation of membrane and secretory proteins that contribute to endoplasmic reticulum stress. Together with high rates of protein translation in proliferating tumors, including translation of mutant and trans-spliced proteins, leads to further and frequent misfolding of proteins in the TME [173]. Proper protein folding is disturbed by an accumulation of reactive oxygen species (ROS). As cancer cells utilize glycolysis for their metabolic activities, and thus produce large amounts of lactic acid, the pH in the TME is very low, triggering the stress pathway known as the unfolded protein response (UPR) [173–176]. Activation of the UPR leads to both ROS production and dysregulated cytosolic and ER calcium levels [176–178]. Additionally, nutrient deficiencies, notably, limited glucose and glutamine are common in solid tumors [177, 179]. This deprivation leads to disruption in the N-linked glycosylation and folding of proteins in the ER. Limitations in glucose additionally reduce activity of the sarcoplasmic/endoplasmic reticulum calcium ATPase (SERCA) pump, resulting in depleted ER calcium stores and enhanced cytosolic calcium [173, 179].

In addition to the calcium-centric mechanisms of PS externalization in the cancer microenvironment by TMEM16F, emerging expression data from the TCGA public sets also suggests that other types of calcium-dependent and calcium-independent scramblases can be overexpressed. For example, extracellular ATP levels in the TME are much higher than those in normal tissue (Fig. 7D). This ATP binds P2X7 receptors, known to be overexpressed in multiple forms of solid cancers [168, 180, 181]. Activation of the P2X7 receptor and its coupling with the membrane protein EROS leads to PS externalization, as well as cytosolic calcium influx [182]. The mechanism through which P2X7 externalizes PS requires further study, but presents an ATP-dependent, TMEM16F-independent system through the scramblase Xk complexed with Vps13a [182].

Although the detailed mechanisms by which tumor cells dysregulate calcium and in turn contribute to PS externalization are still emerging, interesting proof-of-concept studies by Wang and colleagues established “a PS out” tumor model by targeting the PS flippase component CDC50A. This study supports the idea of a cell intrinsic role for PS on solid cancers in the context of the complex in vivo tumor microenvironment [183]. Artificially, these cells constitutively externalize PS on viable cells and recapitulate the features associated with the tumor microenvironment. When “PS out” MC38 colon adenocarcinoma cells were flank implanted into immunocompetent mice, but not RAG (-/-) NSG mice, the tumor growth was potentiated only in the immunocompetent mice. Moreover, at the immune level, such tumors showed down-regulated MHC class I and MHC Class II, higher M2/M1 macrophage ratios, and a less expanded antigen-specific CD8 + T cell response, all consistent with an overall immune suppressed microenvironment. At the molecular level, PS-out tumor cells engage inhibitory PS receptors including TIM-3, supporting the notion that PS viable cells can contribute to the strong immune-inhibitory responses *analogous to Xkr8*. A conceptually similar study has been reported for ATP11B, whereby tumors with low or absent ATP11B in conjunction with high de novo levels of PS biosynthesis (high PTDSS2; PS-S2) were associated with poor prognosis and the accumulation of myeloid suppressor cells and reduced activity of cytotoxic T cells [184]. This idea is consistent with the observations that the amount of externalized PS in cancer cells is variable depending on the net activities of scramblases and flippases, as well as in the de novo synthesis of PS [185]. As noted above, further research is needed to explore the role of oncogenic kinases that phosphorylate Xkr8 as well as sporadic mutations in flippases that can drive PS externalization (Fig. 7E, F).

While the aforementioned PS-out approach using either a CDC50 flippase engineered cell or the ATP11B negative cells provides an important conceptual rationale for the PS exposure on tumor cells as a cell intrinsic mechanism that drives immune escape, an equally important aspect of dysregulated PS in the tumor microenvironment that still requires better understanding is how tumor cells, versus tumor associated stroma cells, vascular cells, or immune cells (or both) contribute to the totality of constitutive PS externalization in solid tumors. For example, in the original papers that described Bavituximab, a PS-targeting monoclonal antibody that was investigated mechanistically in mice, PS targeting was not only noted on tumors, but also the tumor vascular cells [186] and activated monocytes including DCs and macrophages. On macrophages, in vivo PS-targeting antibodies repolarized subsets towards M1 and substantially increased the M1/M2 ratios in the tumor microenvironment [187]. Concomitantly, in vivo PS-targeting antibodies resulted in maturation of DCs and increased T cell infusion into tumors. Clearly, the ability to use CITE-seq to identify the cell types and dynamics of PS externalization in the tumor microenvironment would not only better understand PS externalization, but also the mechanisms by which PS-targeting antibodies and biologics provide therapeutic utility and efficacy.

### Constitutively dysregulated PS is an agnostic and universal therapeutic target in cancers

**Fig. 8:**
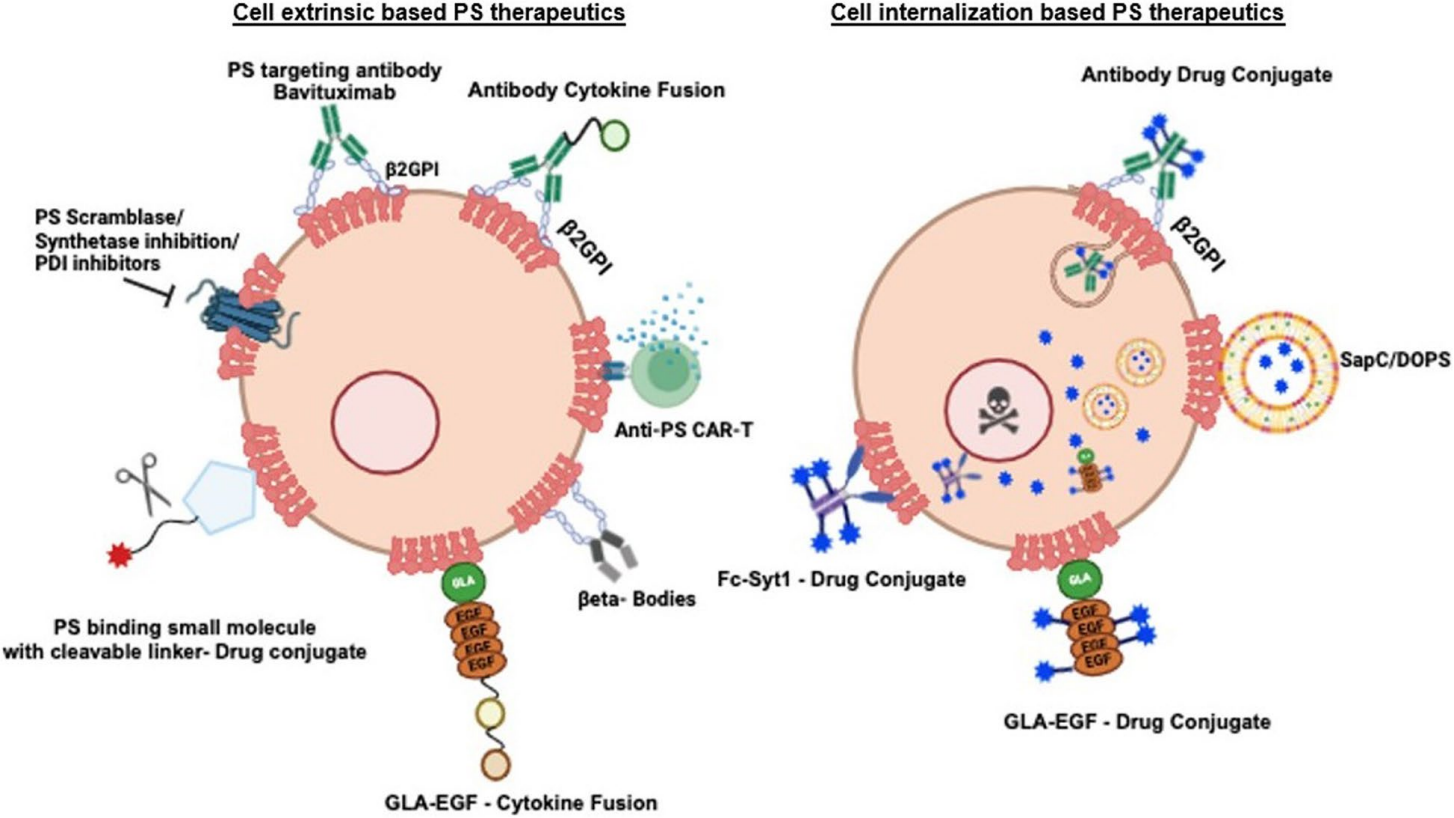
Strategies to target externalized PS in cancer. PS can be targeted both intrinsically and extrinsically to achieve an antitumoral immune stimulatory outcome. Cell extrinsic therapies (left) include modalities that bind to cell externalized PS as well as modalities to inhibit PS scrambling. They are proposed to reduce or nullify the ability for PS to polarize the TME (or reduce the quantity of PS overall) as well as deliver therapeutics with PS as the homing beacon to the TME. Cell intrinsic methods (right) may become internalized within the cell upon PS binding to deliver intracellular therapeutics

The maintenance of PS asymmetry under homeostatic physiological conditions and tight regulation of externalization during apoptosis and physiological activation versus the ensuring chronic and pathophysiological externalization in solid cancers clearly predicts chronic PS externalization as a disease marker and promising therapeutic target [59]. The notion that tumors and the tumor microenvironment represent a unique niche for constitutive and chronically externalized PS is supported by a number of distinct types of PS-binding modalities hone in and target the solid tumor microenvironment as a common observation (Fig. 8). These include PS-targeting monoclonal antibodies such as Bavituximab, 1N11, 11.31, recombinant PS binding proteins such as Annexin’s, beta 2 glycoprotein nanobodies [188], and direct chemical PS binding entities such as SapC-DOPS, the latter induces caspase-dependent apoptosis and bypasses the immune responses of PS targeting antibodies [189, 190]. The fact that disparate types of PS-targeting modalities are able to target and localize to the tumor microenvironments adds credence to the universality of PS as a universal targeting antigen. Below, we highlight the historical and more recent novel strategies by which PS-targeting modalities might be considered for therapeutic benefit to enhance host immunity.

### Targeting PS and PS-R in cancer with PS-targeting antibodies and neutralizing antibodies to TIMs and TAMs

Historically, the first efforts to target PS in the tumor microenvironment centered on the characterization of recombinant high-affinity binding Annexins injected into tumor-bearing mice. Studies by Bondanza and colleagues showed that AnxV, which binds with high affinity to PS, could preferentially target irradiated lymphoma cells and enhance their immunogenicity in a cancer vaccine model, suggesting that endogenous adjuvants binding dying cells could be therapeutically exploited [191]. Based in part on these observations, separate strategies to target PS as an immune escape mechanism centered on the production of a series of PS-targeting monoclonal antibodies aimed to target constitutively exposed PS on the vascular of viable tumor endothelial cells as on hypoxic viable tumor cells and tumor stem cells [187, 192–196]. Bavituximab is a chimeric monoclonal antibody that functions as a checkpoint inhibitor against phosphatidylserine. It was developed by the lab of Philip E. Thorpe at the University of Texas Southwestern Medical Center. Its patent was acquired by Peregrine Pharmaceuticals and in preclinical studies, it showed inhibited tumor growth, prolong survival, and synergistically enhanced the efficacy of concomitant chemotherapy and radiation [193]. Subsequently, Peregrine patents were acquired by OncXerna Therapeutics and now by Feng Biosciences.

While the use of Bavituximab, terminating in a failed phase 3 clinical trial in non-small cell lung cancer (NSCLC) called the SUNRISE trial, is not without caveats, studies have not ruled out the utility of PS-targeting Mabs in other applications. First, the design of the SUNRISE trial utilized Bavitiximab with Docetaxol (Taxotere) a chemotherapeutic agent, in patients with previously treated advanced non-small-cell lung cancer (NSCLC). Patients in this study received up to six 21-day cycles of docetaxel plus weekly bavituximab at 3 mg/kg or placebo until progression or toxicity. The primary endpoint of this study was to assess the overall survival of these patients. This study was halted after assessment of preliminary data showed no benefit between the patients bavituximab group against the placebo [197]. As noted, several caveats of the Bavituximab trial are noteworthy. First, the patients who received Docetaxol alone outperformed the historical standard of care of Docetaxol. Second, Docetaxol (and Taxols and Taxanes in general) do not induce immunogenic cell death, unlike anthracyclines such as doxorubicin and daunorubicin, which are more likely to induce a host anti-tumor immune response. Interestingly, a post hoc analysis of the SUNRISE trial showed that patients who received Bavituximab and Docetaxel and subsequently received checkpoint inhibitors as the next line of therapy showed significantly improved overall survival, suggesting that the combination of Bavituximab and Pembrolizumab (PD-1 inhibitor) could be clinically beneficial for patients [198]. This post hoc analysis added interest to Bavituximab, and several clinical trials were initiated with a combination of Bavituximab and Pembrolizumab [199]. Historically, it is rare to see a new drug entity fail after a single clinical trial. For example, anti VEGF/ Bevacizumab failed over 10 clinical trials before a successful application was determined for macular degeneration. Currently, there are still at least 4 phase II studies initiated for different types of cancer such as prostate cancer, pancreatic cancer, breast cancer, advanced unresectable hepatocellular carcinoma, and advanced non-small-cell lung cancer (NSCLC) [199]. with some still reporting encouraging outcomes [200].

Analogous to the aforementioned PS-targeting mAbs alluded to above, several efforts have been made to block PS receptors on macrophages, monocytes, and T cells. Most emblematically, these efforts have mainly focused on TAM and TIM receptors, as discussed above. With respect to targeting TAMs (Tyro3, Axl, and Mertk), while all three TAMs bind indirectly to PS via their ligands Gas6 and Pros1, the studies with Mertk appear most promising and the rationale for this is severalfold. First, Mertk appears to be primarily expressed on M2c and tumor associated macrophages, and clearly plays a dominant role in efferocytosis. Second, several studies have shown that Mertk can act as a PS sensor or rheostat, whereby the intensity of Mertk signaling is related to the concentration of PS. Indeed, both genetic and pharmacological models suggest that Mertk may act as a myeloid checkpoint, possibly akin to PD1 on T cells. Adding diversity and versatility, other types of inhibitors, including receptor traps, non-carboxylated ligands, and degraders add to this research and therapeutic field [201–205], and clearly, it will be interesting to see how these agents perform in pre-clinical studies. It has been observed in colorectal cancer patient samples that there is an upregulation of both Gas6 and TAM receptor, Tyro3, compared to non-diseased tissue [206].

The above-mentioned inhibitory role for Mertk on myeloid cells and macrophages has led in recent years to considerable ferment in the area of cancer immunology aimed to target Mertk akin to the so-called myeloid checkpoint inhibitors. For example, the Genentech group has shown that treatment of tumor-bearing mice with a highly selective anti-Mertk neutralizing antibody impairs macrophage efferocytosis, resulting in the release of danger signals such as ATP and cGAS that activate macrophages to help stimulate host immunity to cancer cells [207]. Similarly, using neutralizing anti-Mertk antibodies developed by Bristol Myers Squibb, targeting Mertk on macrophages synergized with radiotherapy in improving host anti-tumor immunity towards lung adenocarcinoma in tumor bearing mice [208], as well as improved host anti-tumor immunity in triple negative breast cancer models [95]. Such models suggest that blocking Mertk as a PS receptor for efferocytosis on macrophages may promote secondary necrosis and the beneficial immune effects of inflammatory cell death signals associated with immunogenic death or programmed necrosis/necroptosis. This idea is further supported by interesting studies by Sekar and colleagues showing that depletion of Phosphatidylserine Synthase 1 (PTDSS1) from tumor cells suppressed tumor growth and reduced the numbers of tumor associated macrophages, an observation that could also be phenocopied by knockout of Mertk [209].

### Emerging strategies in PS targeting modalities

**Fig. 9:**
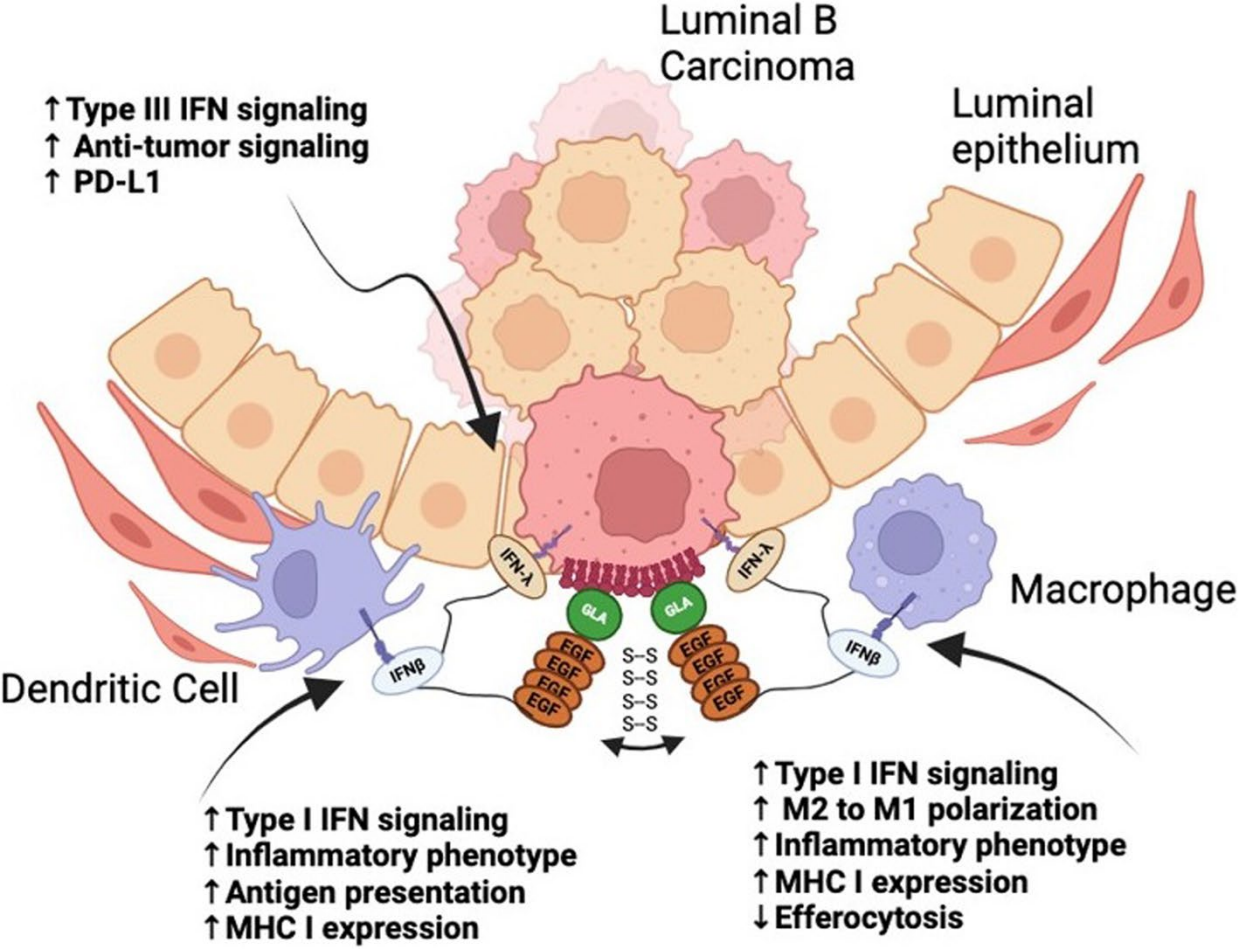
Fusion of PS-targeting domains with dual interferons provides a novel immune stimulating payload. Gas6-IFN-β IFN-λ provides a PS targeting modality combined with dual acting IFNs as an immunogenic payload. The IFNβ is expected then bind any nucleated immune cells in the TME, while the IFNλ binds epithelial cells, including the PS exposed on the tumor cells. IFN signaling will then result in an inflammatory, hot tumor phenotype and reduced efferocytosis

While PS and PS-R targeting represent a viable, interesting, and still emerging approach in immune-oncology, recent studies provide rationale and support for targeting the most proximal PS source, namely the PS scramblases target externalize PS during apoptosis (Xkr8) and oncogenic stress (TMEM16F) [59, 210, 211]. Multiple methodologies of tumor targeting have been developed across many labs to localize therapeutics to the tumors via PS. These therapeutic strategies include Gas6 fusion IFNs [212], Gla-mediated targeting [213], PS decarboxylase [214], antibody–drug conjugates (ADCs) [215, 216], CAR-T cells [217–219], and CAR-Macrophages [220]. The Birge lab has developed a novel PS-targeting therapeutic wherein the gamma-carboxylic acid (GLA) domain of the PS-targeting bridging molecule, Gas6, is bound to an interferon duet containing IFNβ and IFNλ fused by a flexible linker (Gas6-IFNβλ) (Fig. 9) [212]. As type I IFNs (IFNβ) bind to all nucleated cells and type III (IFNλ) bind to epithelial cells, this fusion allows for the binding and delivery of the drug to different cell subsets, including tumor and immune cells within the tumor microenvironment [221, 222]. Gas6 allows for the localization of the therapeutic to the tumor site, using PS as a homing beacon, and the subsequent delivery of the proteins to the TME. This molecule allows for a more targeted approach to delivering immunotherapies, reducing the adverse off-target effects by localizing to the tumor-externalized PS [212]. Another variation of targeting PS involves the use of the GLA domain of Protein S, another PS-binding protein, to target the PS-externalizing cells within the TME. The Contag lab, in collaboration with Gladiator Biosciences, has shown that these molecules bind externalized PS and are then internalized into the cells. This renders the molecule promising as a drug delivery method for anti-tumor therapeutics as well as for any other pathogenic conditions known to externalize PS [213].

Beyond the use of Protein S and Gas6, an antibody-based delivery system localizes an anti-tumor drug to the TME without the adverse effects of whole-body treatment exposure [223]. In a PS-based context, multiple different PS targeting molecules have been utilized in this therapeutic modality in the hopes of delivering a tolerable drug dose to the tumor site specifically, including zinc(II) dipicolylamine [215] and Annexins [216]. PS decarboxylase (PSD) has also been explored as a modality of targeting the PS pathway aside from using Gas6 and Protein S. This enzyme is responsible for the degradation of PS, which then feeds into the synthesis of PE [224]. Therapeutics such as Doxorubicin have been observed to inhibit PSD, leading to reduced PE levels, decreased OXPHOS efficiency, and resulting in cell death [214]. This presents a PS-based approach wherein the enzyme that decarboxylates PS can be exploited and inhibited to promote tumor cell death.

Chimeric antigen receptor (CAR) therapies have been developed to fight multiple forms of cancer through the use of the patient's own T cells, NK cells, or macrophages. CAR therapies allow a patient’s blood cells to be extracted and the desired immune cell subset to be isolated. These cells can then be engineered to target the patient’s tumor and reintroduced into the body [225]. CAR-macrophages have been developed containing portions of MerTK, a PS-binding tyrosine kinase (RTK) that induces efferocytosis upon receptor binding [220]. CAR-T cells targeting Axl receptors, PS binding RTK receptors, overexpressed in cancers, are currently in clinical trials [217, 219]. In pancreatic cancer, CAR-T cells with the extracellular domain of Gas6 have been seen to be highly effective in killing TAM-positive tumor cells in vitro and in mouse xenograft models [218]. Overall, there have been several recent developments in PS-targeting tumor therapeutic strategies with promising data for low toxicity and off-target effects, and high efficacy.

### Apoptotic mimicry and evolutionary exploitation of PS on enveloped viruses

**Fig. 10:**
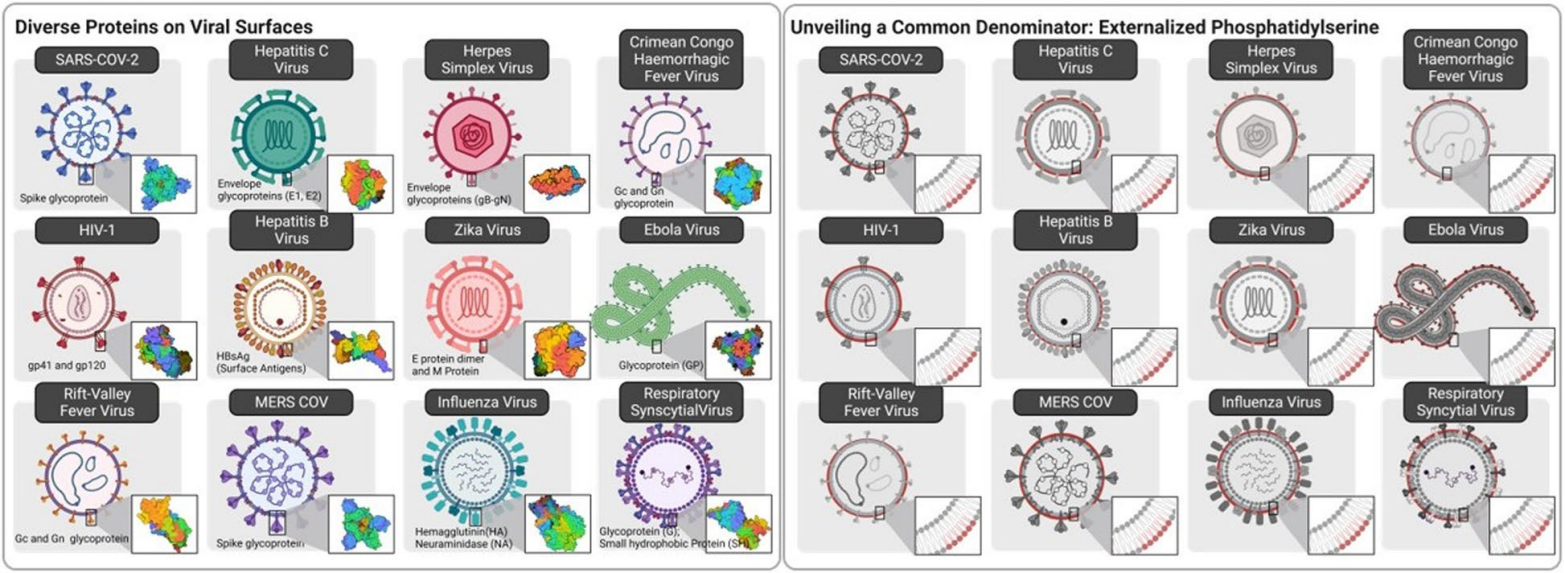
PS externalization on the surface of enveloped viruses and convergent evolution. While viral surfaces display a multitude of different cell surface proteins on their surfaces most, if not all, enveloped viruses share a common characteristic of PS externalized on the surface called apoptotic mimicry. Conceptually, apoptotic mimicry supports the generalized idea that PS represents a universal immunosuppressive mechanism to divert host immunity and that PS is also a promising option to target for antiviral therapeutics

The arguments above that dysregulated PS represents a marker for solid cancers and not normal healthy tissues predicts a vulnerability in cancer that can be exploited therapeutically as an anti-cancer and immunotherapeutic strategy. The rationale for PS as a vulnerability that can be therapeutically exploited is teleologically supported by the profound convergent evolution that has emerged showing enveloped viruses employ to exploit PS as a pathophysiological strategy to both gain entry into cells (using surrogate PS receptors for internalization) as well as immunologically subvert host anti-viral immunity. Shown in Fig. 10, and Table 1 is a partial list of enveloped viruses that have been demonstrated to utilize PS externalization and apoptotic mimicry as a strategy to evade immune detection and increase the likelihood for successful replication. Such convergent evolution, whereby different phylogenetic viruses use a similar strategy, is clearly difficult to ignore in the context of PS and cancer. Indeed, in other scenarios, several viruses converge to target p53, such as adenovirus E1B 55 Kd protein [226], HPV virus E6 protein [227], SV40 Large T antigen [228], and Hepatitis B virus HBx protein [229]. Such convergent evolution provides compelling evidence supporting the importance of PS as a pathophysiological marker to subvert host immunity and is relevant to the biology of PS in solid cancers.

**Table 1.**
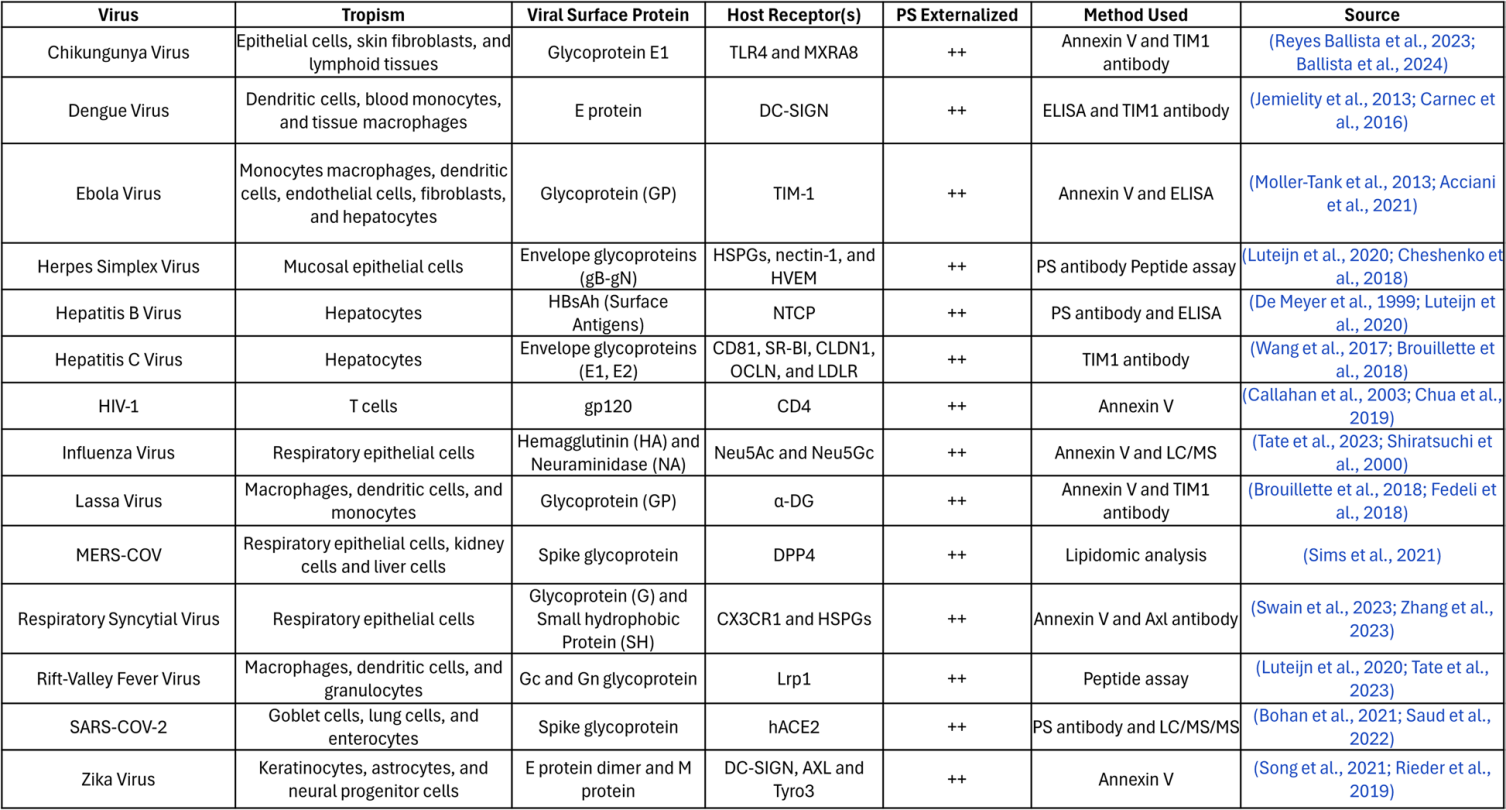
List of enveloped viruses, tropism, relation of protein epitopes and host receptors, and reference to PS externalization by convergent evolution.

## Conclusion and future directions

Historically, the success of cancer therapeutics has been limited by issues related to cancer evolution and problems related to drug resistance. Cancer targets that provide stable and ubiquitous epitopes offer strong potential for translation into future clinical applications. In recent years, a number of strategies have been developed to bind externalized PS in the tumor microenvironment, suggesting that PS is an efficient means of targeting cancer, and that externalized PS plays a role as a cell intrinsic immune escape mechanism in cancer. This notion is strengthened further by the findings that the levels of PS exposure on the outer membrane are particularly high on cell surfaces exposed to stress conditions in the tumor microenvironment. However, optimization and modifications are required to enhance the efficacy of PS targeting modalities as well as reduce toxicity and development of drug resistance. Further development of this class of PS targeting molecules is predicted to lead to the next generation of molecules in immune-oncology.

## Supplementary Information

Supplementary Material 1. Fig S1. A Inhibitory motifs in PS Receptors. The length of the cytoplasmic domain of known PS receptors is represented as grey vertical bars. The positions of the Tyrosine residues and ITIM and ITSM motifs are indicated. B. ITIM motifs bind to protein and lipid phosphatases. The paired ITIM motifs bind optimally to the protein phosphatase (SHP-1/2) whereas the uncoupled ITIM/ITSM domain

Supplementary Material 2. Table S1. Sequence of all the cytoplasmic domains of PS receptors.

## Data Availability

No datasets were generated or analysed during the current study.
