# Supplementary figures and images for: Dys-regulated phosphatidylserine externalization as a cell intrinsic immune escape mechanism in cancer
Source: Cell Commun Signal. 2025 Mar 11;23:131. doi: 10.1186/s12964-025-02090-6 (PMC11900106; doi:10.1186/s12964-025-02090-6)

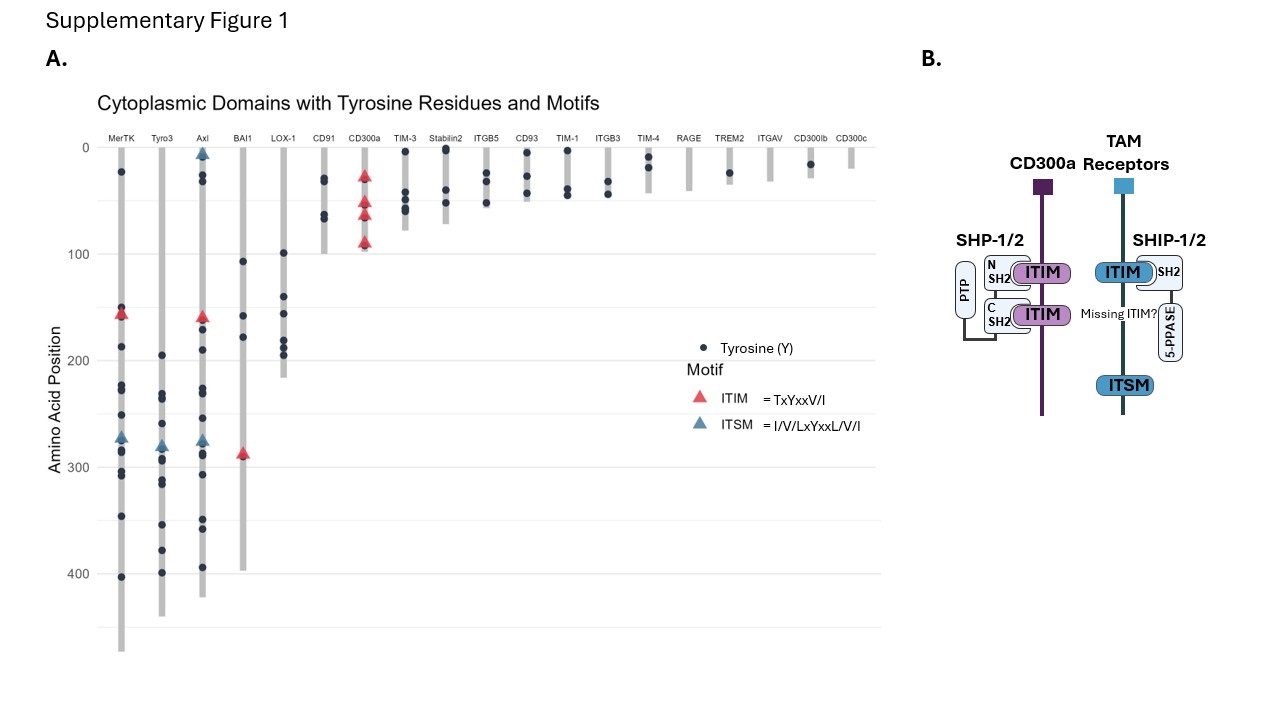

Supplement: Supplementary file 1 — Supplementary Material 1. Fig S1. A Inhibitory motifs in PS Receptors. The length of the cytoplasmic domain of known PS receptors is represented as grey vertical bars. The positions of the Tyrosine residues and ITIM and ITSM motifs are indicated. B. ITIM motifs bind to protein and lipid phosphatases. The paired ITIM motifs bind optimally to the protein phosphatase (SHP-1/2) whereas the uncoupled ITIM/ITSM domain [file 12964_2025_2090_MOESM1_ESM.jpg]

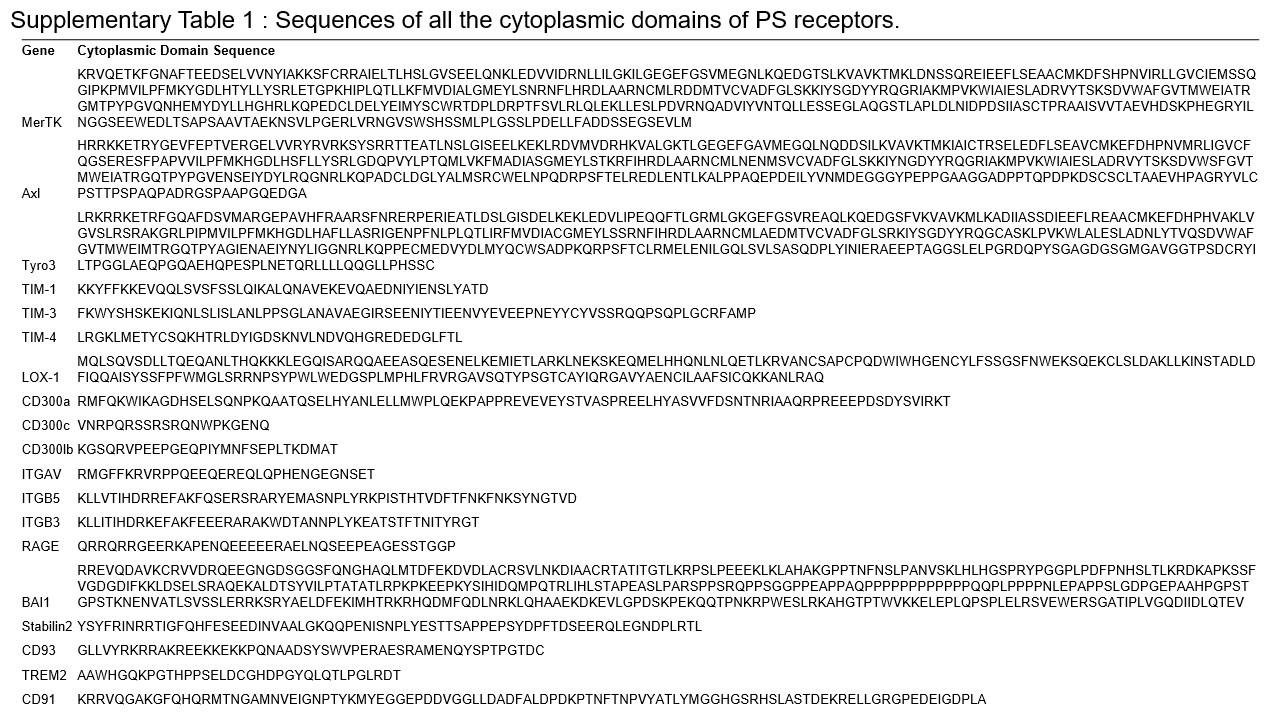

Supplement: Supplementary file 2 — Supplementary Material 2. Table S1. Sequence of all the cytoplasmic domains of PS receptors. [file 12964_2025_2090_MOESM2_ESM.jpg]
